# Roadmap to sustainable plastic waste management: a focused study on recycling PET for triboelectric nanogenerator production in Singapore and India

**DOI:** 10.1007/s11356-022-20854-2

**Published:** 2022-05-23

**Authors:** Wei Liang Lai, Shreya Sharma, Sunanda Roy, Pradip Kumar Maji, Bhasha Sharma, Seeram Ramakrishna, Kheng Lim Goh

**Affiliations:** 1 Newcastle Research & Innovation Institute Singapore (NewRIIS), 80 Jurong East Street 21, #05-04, Singapore, 609607 Singapore; 2 Faculty of Science, Agriculture and Engineering, Newcastle University, Newcastle Upon Tyne, NE1 7RU UK; 3 Department of Biological Sciences and Engineering, Netaji Subhas University of Technology, Delhi, 110078 India; 4 Department of Polymer and Process Engineering, Indian Institute of Technology Roorkee, Saharanpur Campus, Saharanpur, Uttar Pradesh 247001 India; 5 Department of Mechanical Engineering, GLA University, Mathura, Uttar Pradesh 281406 India; 6 Department of Chemistry, University of Delhi, Delhi, 110007 India; 7 Department of Mechanical Engineering, National University of Singapore, Singapore, 117576 Singapore

**Keywords:** Plastic recycling, Policy, Recyclate value model, Sustainability, Triboelectric nanogenerator, Waste management

## Abstract

This study explores the implications of plastic waste and recycling management on recyclates for manufacturing clean-energy harvesting devices. The focus is on a comparative analysis of using recycled polyethylene terephthalate (PET) for triboelectric nanogenerator (TENG) production, in two densely populated Asian countries of large economies, namely Singapore and India. Of the total 930,000 tonnes of plastic waste generated in Singapore in 2019, only 4% were recycled and the rest were incinerated. In comparison, India yielded 8.6 million tonnes of plastic waste and 70% were recycled. Both countries have strict recycling goals and have instituted different waste and recycling management regulations. The findings show that the waste policies and legislations, responsibilities and heterogeneity in collection systems and infrastructure of the respective country are the pivotal attributes to successful recycling. Challenges to recycle plastic include segregation, adulterants and macromolecular structure degradation which could influence the recyclate properties and pose challenges for manufacturing products. A model was developed to evaluate the economic value and mechanical potential of PET recyclate. The model predicted a 30% loss of material performance and a 65% loss of economic value after the first recycling cycle. The economic value depreciates to zero with decreasing mechanical performance of plastic after multiple recycling cycles. For understanding how TENG technology could be incorporated into the circular economy, a model has estimated about 20 million and 7300 billion pieces of aerogel mats can be manufactured from the PET bottles disposed in Singapore and India, respectively which were sufficient to produce small-scale TENG devices for all peoples in both countries.

**Supplementary Information:**

The online version contains supplementary material available at 10.1007/s11356-022-20854-2.

## Introduction

The vast amount of plastics entering the environment annually is contributing to an ever-increasing problem of pollution from plastic waste both on land and in the ocean (Garcia and Robertson 2017). This escalating problem is spurred by the global population growth and demand for plastic products, resulting in the rapid growth in plastic production coupled with the linear ‘take-make-waste’ economy. In addition, on the peak of the COVID-19 pandemic, a substantial increase in single-use plastics, such as personal protective equipment (namely masks and gloves), has further aggravated the plastic waste problem (Lau et al. 2020) .

**Fig. 1:**
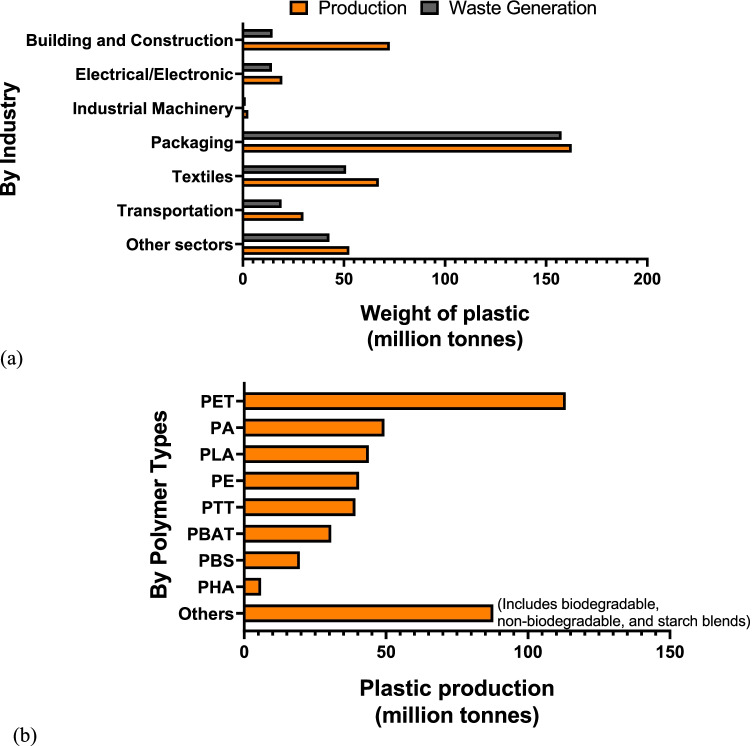
Estimation of global plastic production and waste generation in 2018 by **a** industry and **b** polymer types (Tsakona and Rucevska, 2020)

**Fig. 2:**
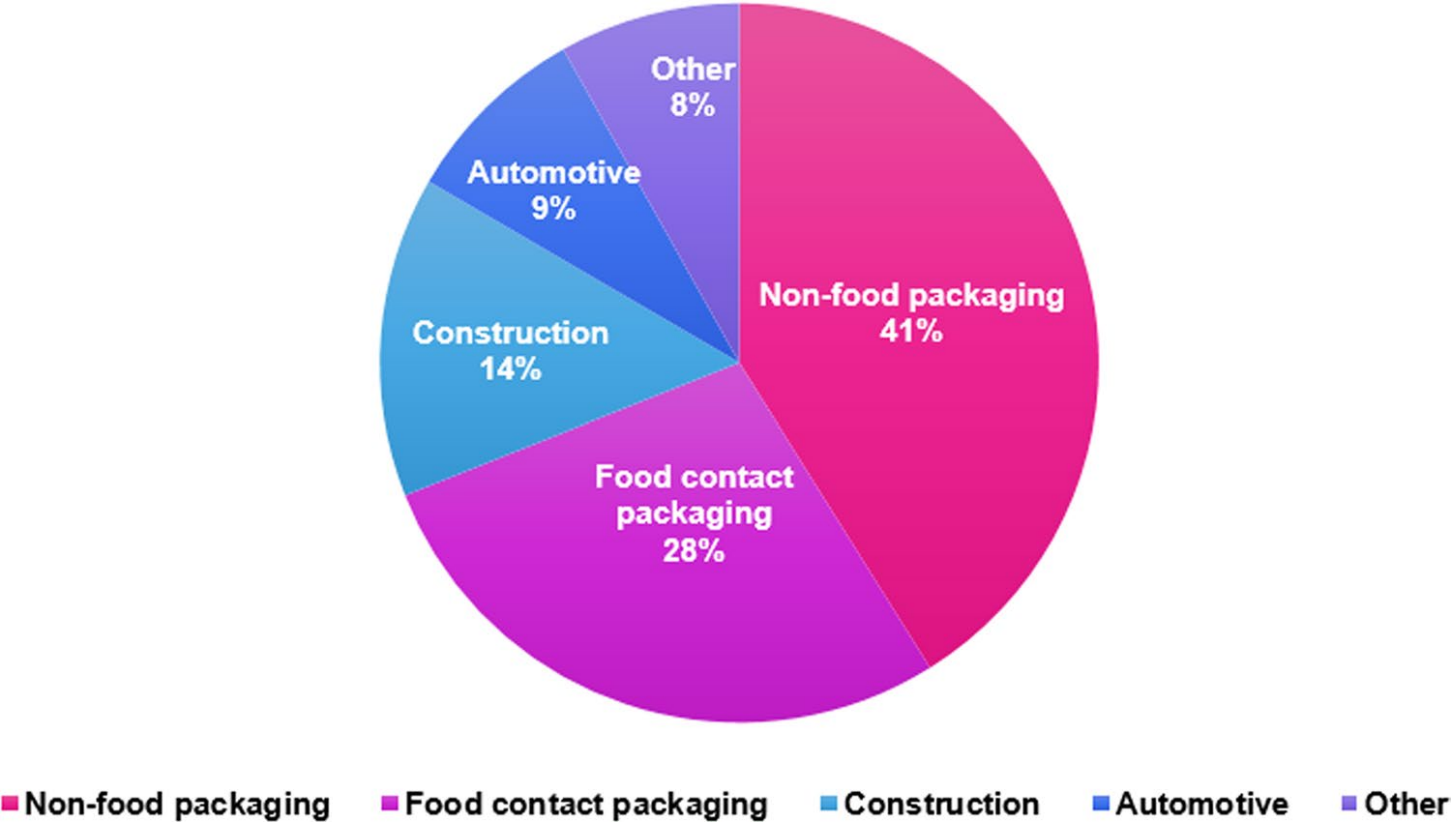
Market share of recycled plastics by application

The pollution and emissions of greenhouse gases and chemical pollutants during plastic production and the clean-up cost of plastic account for US$3.7 trillion in 2019 (more than the GDP of India in 2019) and are projected to reach US$7.1 trillion by 2040 (more than GDP of Australia, Canada, and Germany in 2019 combined) (The Straits Times 2021). Figure 1a and b show an estimation of global plastic production (not based on any particular country) and waste generation with respect to industry sectors and polymer types in 2018, respectively. Commercial production of plastics had outpaced the majority of other manufactured materials reaching ∼ 450 million tonnes in 2018 (Tsakona and Rucevska 2020). Plastic packaging and single-use plastics are the major products from the plastic industry that enters the waste stream almost instantly after usage (see Fig. 1a), contributing to a cumulative total of 6.3 billion tonnes of plastic waste generated worldwide (Joo et al. 2018). The projected increase in the quantity of post-consumer plastic waste comprises a considerable portion of the solid waste stream, attracting reasonable attention in recent years, predominantly in areas of rapid economic development and population growth. The challenge of managing the increasing quantities of plastic debris, especially from short-use products, requires the municipalities to arrange efficient systems for collection, disposal and potential reclamation, recycling and recovery of waste plastics. However, the present-day scenario has accounted for about 40% (by weight) of mismanaged plastic waste (Geyer et al. 2017). It was reported that a small amount of plastics were recycled (about 9%) and incinerated (about 12%), and about 60% of the plastics were discarded to the landfills and natural environment (Geyer et al. 2017). Globally, there is a great urgency to control and reduce unmanaged plastic waste to reduce the pressure on the environment. The global market share of recycled plastics by application is presented through Fig. 2.

Several factors concerning plastic waste management, namely government policies, strategies, accounting behaviour, sustainability indicators and gross domestic product (GDP), have been investigated for possible correlation as reported in many recently published studies (Barnes 2019; Lebreton and Andrady 2019; Cordier et al. 2021). Barnes and co-workers modelled the relationship between the income per capita and mismanaged plastic debris for 151 countries (Barnes 2019). The model predicted that the mismanaged plastic waste per capita increases with increasing income per capita in a country up to a certain level, beyond which the mismanaged plastic waste per capita decreases owing to improved environmental efforts (Barnes 2019). Furthermore, higher capital investment in research and development by higher-income countries provide the means for technological innovations to mitigate the environmental impact of plastic waste (Barnes 2019). It has been proposed that mismanaged waste is inversely correlated to the gross domestic product (GDP) per capita; in other words, an increasing amount of mismanaged waste leads to decreasing GDP (Lebreton and Andrady 2019). A recent study based on the World Bank database argued that improper management of plastic trash was not the only factor; GDP is also dependent on other factors such as geographic location, legislation and policy measures, market regulations and education levels (Cordier et al. 2021). The practice of plastic waste valorization, such as the conversion of plastic waste into fuel, energy and other value-added products, dominates in many developed countries affiliated with the organization for economic cooperation and development (OECD) (Table 1). However, globally, a large proportion of plastic waste was disposed at open dumps or landfills.

**Table 1:** Benefits and drawbacks of plastic waste treatment technologies

Technology	Adoption	Key aspects of advancement	Drawbacks
Common treatment technologies			
Incineration (direct combustion)* (Yang et al. 2021)	All OECD countries (i.e. Belgium, France, Germany, Italy, Netherlands, UK, USA and many more countries)	Energy generated from waste is deemed to be from ‘renewable energy source’ in Europe and America Benefits: 1. Able to reduce the volume of the trash while generating heat and power that can be employed for the generation of electricity 2. An improvement over landfill (Landfill contribute approximately 30% more to global warming.)	1. Prodigious emitter of toxic pollutants such as organic carbon, heavy metal radicals, sulphur dioxide, hydrogen sulphide, nitrogen oxide, hydrogen chloride and carbon monoxide 2. Incineration of plastic waste results in the emission of about 400 million tonnes of CO2 per year
Landfill (Nanda and Berruti 2021)	All OECD countries (i.e. Japan, Iran, Mexico and many more countries)	Utilized as final disposal sites in Japan. Categorized into (1) controlled landfill protected with water shielding sheets for water catchment and seepage and leakage control; (2) inert landfill for biochemically stable waste and (3) isolated landfill for prevention of entry of rainwater and escape of leachate Establishment of the semi-aerobic landfill, also known as a hybrid of aerobic and anaerobic landfills in countries like Japan, Iran, China, Mexico, Malaysia, Thailand and Vietnam for a comparatively sanitary approach against the greenhouse effect Benefits: cheap and affordable way of plastic waste disposal	1. Landfill gas is a major contributor to global warming 2. Leachate (fluid percolating through landfills) pollutes land, ground water and waterways
Mechanical recycling^#^ (Schyns and Shaver 2021)	All OECD countries (i.e. Belgium, France, Germany, Italy, Netherlands, UK, USA and many more countries)	Benefits: 1. Less energy-intensive 2. Manufacturing of recycled materials three times more efficient with respect to greenhouse gas emissions when compared to manufacturing of products from virgin raw materials 3. Flexible feedstock supply with low investment costs 4. Exerts little negative impact to the environment	1. Yield loss during the recycling process 2. Lack of standardization and operational excellence (remains underdeveloped) 3. Quality loss which eventually requires disposal in landfill 4. Thermoset polymer cannot be recycle using this method 5. Coloured polymers need to be variegated and the property of recyclable may deteriorate following reach recycling cycle 6. Intra-molecular crosslinks and oxidation reaction may result in yellowing of the recycled polymer 7. Thermal and hydrolytic digestion reduction in molecular weight (or intrinsic viscosity) is a crucial problem
Thermal treatment technologies			
Pyrolysis^@^ (Antelava et al. 2021; Dogu et al. 2021)	Europe, China	Benefits: 1. Flexible technology to convert plastic waste to beneficial energy products in absence of oxygen 2. Tolerate a higher level of contaminants in the feed and therefore contemplated as appealing in terms of economics because of the limited number of pre-treatments needed upstreaming 3. Simple yet sustainable process as produced energy circulates back into the process 4. Liberty of tuning the desired end-product by varying the different operating conditions, reactor type and utilization of catalyst 5. Liquid oil or wax obtained may be used as back-up power when needed 6. Catalytic cracking offers conversion at lower temperatures which lowers heat demand and economy of the process	1. Not economical because of high energy needs 2. Varying shape and size of plastic trash make it arduous to feed them to the process 3. Necessary to characterize feedstock before recycling as it may contain toxic substances and harmful additives that may escape from the system presenting critical environmental and health imperils 4. Standard testing methods are non-existent 5. Search for an economical, selective, stable, and active enough catalyst is ongoing 6. Complexity of reaction and high energy consumptions concerning thermal cracking
Gasification (Kijo-kleczkowska and Gnatowski 2022)	China, Japan, USA, Canada, South Africa, Korea	State-of-the-art technology established in Canada to convert non-recyclable/rejectable into biofuels aiming to reduce greenhouse gas emissions by more than 60% Benefits: 1. Detailed breakdown of mixed plastics by oxidation to generate fuel gas for multiple applications 2. Production of new plastic products mediated by steam gasification	1. Not economical because of high energy needs 2. Oxygen separation of air for pure oxygen gasification further increases the cost of process 3. Requirement of high feedstock volume to be feasible 4. Purification of product gas before its use
Plasma gasification (Mazzoni and Janajreh 2017; Munir et al. 2019; Zhao et al. 2022)	China, Japan, Taiwan, Canada	Benefits: decomposition of tar, char and dioxins to produce highly pure synthesis gas	High capital investment and electricity consumption
Microwave-assisted pyrolysis/ gasification (Zhang et al. 2017; Suresh et al. 2021)	USA, Korea	Benefits: microwave irradiation offer benefits including augmented production speed, more control over the process and higher heating rates and even distribution	1. Not suitable for mixed waste composition 2. Requires large feedstock volume
Hydrothermal liquefaction (Ahamed Kameel et al. 2022)	South Africa, Europe, Australia, Turkey, Canada, Denmark	Several industrial demonstration projects taken up by different companies worldwide Benefits: 1. Lesser feedstock consumption to produce an energy efficiency of 85–90% 2. Tolerates high moisture content of the feedstock	1. Still at an early development stage and reaction chemistry needs to be understood 2. Requires high pressure working conditions leading to high cost and several technical barriers
Biological treatment technologies			
Compositing (Ayilara et al. 2020)	Europe, USA	Benefits: 1. Minimal carbon emitted 2. Does not produce secondary pollutants associated with incineration and landfilling	1. Still at an early development stage 2. Generate microplastics that contribute to environmental problems
Anaerobic digestion (Quecholac-Piña et al. 2020; Chen et al. 2021)	Europe, Japan, USA, Korea	Benefits: 1. Widely used source for renewable energy 2. Low energy consumption process 3. The biogas produced acts as an excellent source for fuel in heat and power gas engines	1. High-level investment 2. Causes odour nuisance
Bio-recycling (Ramakrishna et al. 2021)	Germany, Singapore	Strategic partnerships between Germany and Singapore for research and development of technology Benefits: 1. New biotechnology and enzymes for plastic degradation and circularization 2. Converting plastic waste into other bio-products and for reintegrating biomass in a safe and sustainable way 3. Low carbon footprint 4. Efficient, organic and low energy method For example, an enzyme that has been identified during the degrading of PET could be converted to terephthalic acid and ethylene glycol	1. High-level investment 2. Causes odour nuisance 3. Require the uses of microorganisms (i.e. superworm *Zophobas morio* for degrading polystyrene)

#See “Mechanical recycling”; @see “Pyrolysis**”**; *see “Incineration”

It is important to emphasize that developed economies depend on waste-to-energy technologies involving techniques such as incineration, pyrolysis, gasification, plasma pyrolysis, plasma gasification, landfill gas utilization, composting, anaerobic digestion with biogas recovery, bio-hydrogen production, bio-recycling and many other technologies to create a real circular economy (Munir et al. 2021).

The intent of this report is to assess the implications of plastic waste management and recycling regulations on the recyclates for manufacturing advanced clean-energy harvesting devices. In particular, the focus is on a comparative analysis of the use of recycled polyethylene terephthalate (PET) for triboelectric nanogenerators (TENGs), in two densely populated Asian countries of large economies by GDP, Singapore and India. Here, two seemingly disproportionate Asian countries stand out: India and Singapore (Lebreton and Andrady 2019). The land area of India is about 4516 times larger than Singapore, and the population of India is about 243 times bigger than Singapore. A summary of the comparison of the land area, population, population density and GDP between Singapore and India were presented in Table 2. In 2019, Singapore, a high-income, high-density small city-state in Asia, with the 4th highest GDP per capita ranking in the world (Worldometers GDP per Capita n.d), generated a total of 930 thousand tonnes of plastic waste, with only 4% recycled (National Environment Agency 2021a). In comparison, India, a low-income, a highly populated country in Asia, generated a much larger tonnage of plastic waste, at 8.6 million tonnes. Yet, India could achieve a 70% recycling rate (Central Pollution Control Board 2019; Ministry of Housing and Urban Affair 2019). Singapore relies heavily on the incineration of plastic waste to satiate the energy needs of a robust, growing industry, and therefore, it becomes important to study renewable energy alternatives which could aid in minimizing the electricity generation cost while promoting cleaner production. At the same time, India is perceived to have an enormous potential for adopting the waste-to-energy practice, largely because of the high population and its inclusion in the group of emerging countries that will endure an upsurge in waste generation and energy consumption shortly.

**Table 2:** Land area, population and GDP of Singapore and India

	Singapore	India	Scale
Land area (km^2^)	728	3,287,590	1:4516
Population	5,685,807	1,380,004,385	1:243
Population density (km^2^)	7810	420	-
GDP	USD 339,998,477,000 (USD 59,798 per capita)	USD 2,622,984,000,000 (USD 1,900 per capita)	1:8 (31:1)

The present study aims to understand the current plastic waste management practices from the perspective of countries in South Asia (India) and South-East Asia (Singapore). The study evaluates the exacerbating problem of plastic waste and the management of plastic waste based on the existing regulations and policies. Typically, about 43% and 33% of the total plastic waste produced were utilized for packaging purposes in India (Kapur-Bakshi et al. 2021) and Singapore (Jen Teo 2018), respectively. Of particular interest here is polyethylene terephthalate (PET), a polymer widely utilized in the packaging industry (see Fig. 1b) due to its high specific modulus (modulus to weight ratio), low cost, ease of processing, durability, high resistance to heat and chemicals, hydrophobicity and bio-inertness (Geyer et al. 2017; Lebreton and Andrady 2019). Recycling methods such as sorting, washing, grinding, and extrusion processes can facilitate up to 37% and 90% of PET waste in Singapore (Singapore Environment Council 2021) and India (NCL Innovations, CSIR-NCL 2013; Plasteurope.com 2017), respectively. Unfortunately, the mechanical and structural properties of the recycled products were found to deteriorate progressively with each cycle (Alvarado Chacon et al. 2020). The reduction in properties were attributable to the thermo-mechanical and thermo-oxidative degradation of the polymeric chains coupled with hydrolytic scission. Herein, a summary of different studies (Table 3) report on the degradation of mechanical and structural properties of recycled commodity polymers. Therefore, it becomes important to assess the material value sustainability and material cost impact with respect to the number of recycling cycles and industry applications (Brouwer et al. 2020).

**Table 3:** Degradation in properties of commodity polymers post-recycling reported previously in the literature

Reference	Recycled polymer	% degradation in properties
Dahlbo et al. (2018)	Polypropylene	26% yield strength, 86% elongation, 21% modulus
Cress et al. (2021)	Acrylonitrile butadiene styrene	10% tensile and fracture strength, 25% strain at break, 37% toughness
Budin et al. (2019)	Polylactic acid	11% tensile strength, 5% transverse rupture test, 50% impact strength, 4% hardness
Mendes et al. (2011)	High-density polyethylene	40% crystallinity degree
Eriksen et al. (2019)	Polyethylene terephthalate	56% tensile strength

In this study, a circular economy approach was introduced for managing PET and its waste. A model was developed to predict the raw material cost and mechanical integrity of the recycled PET materials. In view of the increasing energy consumption by the respective countries, putting pressure on the production of energy, this study explores the pathway leading to the mass manufacturing of the triboelectric nanogenerator (TENG), a novel energy-harvesting device that has the potential for every day-use, from recycled PET materials. First reported in 2012, TENG is a promising technology for harvesting the mechanical energy of the human body and environment and transforming into electrical energy (Wang et al. 2012, 2017b). The benefits of TENG include low cost, simplicity to manufacture, high output performance at low frequencies and unrestricted material selection (Wang et al. 2017b). Altogether, these benefits were exploited to design intelligent sensing and miniaturized portable harvesters to charge low-power electronics (Zi et al. 2016). In a study published recently, recycled PET was used to make TENG with little loss of performance (Roy et al. 2021). Here, the TENG performance, benefits and limitations from several studies were also reviewed. The implications on the economic material value of using recycled PET for TENG production were explored. An order of magnitude estimate was provided for the simplest application, i.e. an everyday wearable application namely the digital wrist watch (as an alternative to existing solar-powered ones) (Wang et al. 2017a). It might be argued that the choice of the application could be expanded to other applications, such as hearing aids and even contact tracing devices (Yang Boon 2021) that were used for monitoring COVID-19 infections in the community. The waste-to-energy (WTE) treatment were argued to be not suitable for recovering the resources when the ‘reduce, reuse and recycle’ (3R’s) methods could be implemented.

This article presents the findings of the plastic waste management policies, legislation and infrastructure of both Asian countries (India and Singapore) that highlights the proper management and mismanagement of plastic waste and their environmental pollution and energy consumption. Further to address the gaps and add to the body of knowledge, this article focuses on the progress of TENGs for renewable energy generation by recycling PET plastic (for increasing recycling rate) for the manufacturing of triboelectric material. The article aims to provide a perspective to understand plastic waste as an evolving resource that could be proposed and implemented in the waste-to-energy technology for improving plastic waste management in both countries.

## Landscape of plastic waste management and recycling

This section discussed the current legislation and policies for plastic waste management and recycling, related infrastructure and recycling statistics in Singapore and India. The legislation in waste and recycling management has been constantly amended by the governing body in the respective countries (Singapore and India) to provide an opportunity to implement new regime. The related infrastructure and processes facilitate the flow of plastic waste and recyclables, and the statistics of the total plastic waste generated and the recycling rate of the respective countries were presented.

### Legislation and policies

Table 4 shows the comparison of the plastic waste management regulations and goals of Singapore and India. In Singapore, the National Environment Agency (NEA) administers licencing and regulatory functions of waste collection, treatment and disposal (National Environment Agency 2021b). The Environment Public Health Act (EPHA) regulates the waste management system aided by (1) general waste collection regulations, (2) general waste disposal facilities regulations and (3) toxic industrial waste regulations (National Environment Agency 2021b). In 2014, a mandatory waste reporting (MWR) framework was introduced under the EPHA, requiring the owners, occupiers or lessee of a workplace to furnish data on the specific types of waste generated and recycled, and corresponding reduce, reuse and recycle (3Rs) plan annually to the NEA Waste and Resource Management System (WRMS) (National Environment Agency 2021c). NEA states that the purpose of the MWR framework is to bring awareness to the producers on the amount of waste being generated at their premises and also to encourage them to improve their waste management system (National Environment Agency 2021c). Since the enactment of MWR frameworks, NEA has found that more commercial premises have step up their effort in the recycling activities which had led to an increased in the recycling rate in 2019, e.g. 7.4% and 11.4% for the respective hotels and malls (National Environment Agency 2021c).

**Table 4:** Comparison of waste management regulations and goals between Singapore (National Environment Agency 2021b, c) and India (Ministry of Environment 2016a; Pani and Pathak 2021)

	Singapore		India
Governing body	Ministry of Sustainability and the Environment (MSE). Responsible for improving and sustaining a clean environment, promoting sustainability and resource efficiency and maintaining high public health standards		Ministry of Environment, Forest, and Climate (MoEF). Responsible for planning, promoting, coordinating and overseeing the implementation of environmental and forestry programmes in India
Regulatory level	National and municipal • National: NEA is a statutory board under MSE. NEA formulates policy and administers licencing and regulatory functions of waste collection, treatment and disposal • Municipal: town council manage and maintain the common property of the public housing estate (which includes managing of waste and recyclables located at the common property such as void decks, common corridors and lift areas.)		National, state and municipal • National: CPCB is a statutory organization under MoEF. CPCB promotes cleanliness in different areas of the States by prevention, and control to improve the environmental conditions in the country • State: SPCB implements environmental laws and rules within the state’s jurisdiction and to raise awareness to its residents regarding sustainable development to improve environmental quality • Municipal: municipal authority plan, implement and monitor all systems of urban service delivery of municipal solid waste
Legislation	Environment Public Health Act (EPHA), Chapter 95, Revised 2002 • Enforcing environmental health responsibilities by creating a standard code for health-related issues (such as public cleaning, markets, food establishments, general health, sanitation and hygiene) • To strive for higher cleaning and hygiene standards to address waste disposal into streams, rivers, canals, drains, reservoirs, lakes and catchment areas Resource Sustainability Act 2019: • To regulate the Mandatory Packaging Reporting (MPR) framework • To strategize for packaging reduction		Plastic waste management (PWM) rules under the Environment protection act, 1986 • Enforcing rules to protect and improve environmental quality, control and reduce pollution from all sources, and prohibit or restrict the setting and/or operation of any industrial facility on environmental grounds • To raise the producer’s awareness of the amount of waste produced and to improve waste management
Framework	MWR framework: to raise awareness on the amount of waste produced by manufacturers, and to improve waste management		PWM Rules Extended Producer Responsibility (EPR) framework: to monitor packaging data and to validate the effectiveness of PWM rules by CPCB
Plastic waste collection	Licenced public waste collectors collect the general waste and recyclables using dedicated truck and deliver it to the Waste-to-energy (WTE incineration facilities and the Materials Recovery Facilities (MRFs)), respectively		Gram panchayats and Municipalities oversee the collections and segregations of general waste and recyclables. Heavily depends on informal waste collectors including, trash/rag pickers and road sweepers, Dhalaos (dumping spot), intermediate dealers/junkyard owners, and recyclers
Goals			
• To strengthen climate resilience, resource resilience and economic resilience by adopting the circular economy approach • To prolong the lifespan of the Semakau landfill beyond 2035 by reducing the amount of waste sent to landfills by 30% (per capita per day) by 2030 • To minimize the amount of waste by inventing, innovating, and encouraging the uses of waste (e.g. NEWs and for construction, and Magorium for roads) • To achieving beyond 70% of the total recycling rate by looping the plastics in the circular economy		• To address social and environmental challenges and aim to decouple virgin feedstock by adopting the circular economy approach • To reduce landfill/dumping sites due to high population density near the sites which causes stench and affects the living condition • To minimize the amount of waste by inventing, innovating and encouraging the uses of waste (e.g. roads, bricks and other bio-based materials) • To encourage to reuse and recycle of plastics as feedstock in the circular economy without leakages	

In Singapore, typically, one-third of the total generated waste comprises packaging waste (including plastics). Packaging waste has been identified as the key priority waste stream in the Zero Waste Master plan that requires attention and efforts to close the resource loop for Singapore to achieve its goal as a Zero Waste Nation (National Environment Agency 2022a). The enactment of the Resource Sustainability Act (2019) requires the brand owners, manufacturers, importers and retailers (i.e. supermarkets) of packaged products to comply with the Mandatory Packaging Reporting (MPR) framework entailing the reporting of the packaging data (namely material types, form and weight) and 3R strategies (including initiatives, plans, goals and key performance indicators for tracking the progress of plastic reduction in subsequent years) to the WRMS. The Act was fully enforced on the 1st of January 2021 (Republic of Singapore Government 2022). The MPR framework aims to curb the utilization of single-use plastics and improve the plastic recycling rate (Jen Teo 2018; National Environment Agency 2022a).

In India, the Ministry of Environment, Forest, and Climate change (MoEF) in conjunction with the pollution control boards (Central Pollution Control Board (CPCB) and State Pollution Control Board (SPCB)) regulates the waste management system. In 2011, the MoEF enacted the Plastic Waste (management and handling) Rules to enforce coercive laws to mandate the responsibilities of the respective SPCBs, urban local bodies and gram panchayats for the imposition of provisions for plastic waste collection, treatment and disposal (Ministry of Housing and Urban Affair 2019). In response to the global concerns on sanitation and waste management through the Swachh Bharat Mission (SBM) dated October 2014, new plastic waste management rules, namely the ‘Plastic Waste Management (PWM) Rules, 2016’, superseded the ‘Plastic Waste (management and handling) Rules, 2011’ (Ministry of Housing and Urban Affair 2019). The new PWM legislation integrates with the concept of the EPR framework to dictate brand owners, importers and producers to establish systems for the assemblage of waste generated from their products within a period of 6 months and subsequent implementation within 2 years. Mandatory registration of manufacturers to sell plastics to user (or members of the public) and transporting plastic waste recyclables to register recyclers are required. Also, individuals involved in recycling waste processes are required to submit a grant application or renewal of registration for recycling. In conformity with the PMW rules, the Solid Waste Management Rules mandated the responsibility of waste producers such as event organizers and institutions to implement waste generation and minimization, prevent illegal disposal of waste and facilitate waste storage following segregation and handing over of the segregated waste to local bodies or agencies authorized by the local bodies. The latest amendment of the rules, referred to as the PWM (Amendment) Rules, 2018, includes a central registration system for brand owners, importers and producers under the purview of CPCB (Ministry of Environment 2016a). Similarly to the MPR framework in Singapore, the advent of the Uniform Framework for EPR in India in June 2020 requires packaging brand owners, importers and producers to submit quarterly reports, while the producer responsibility organizations (PROs), SPCBs and CPCBs submit their annual reports on waste collection and disposal to the National registration and database repository web portal. These reports are intended to inform CPCB for monitoring the effectiveness of the PWM rules (Pani and Pathak 2021).

### Plastic waste management infrastructure

**Fig. 3:**
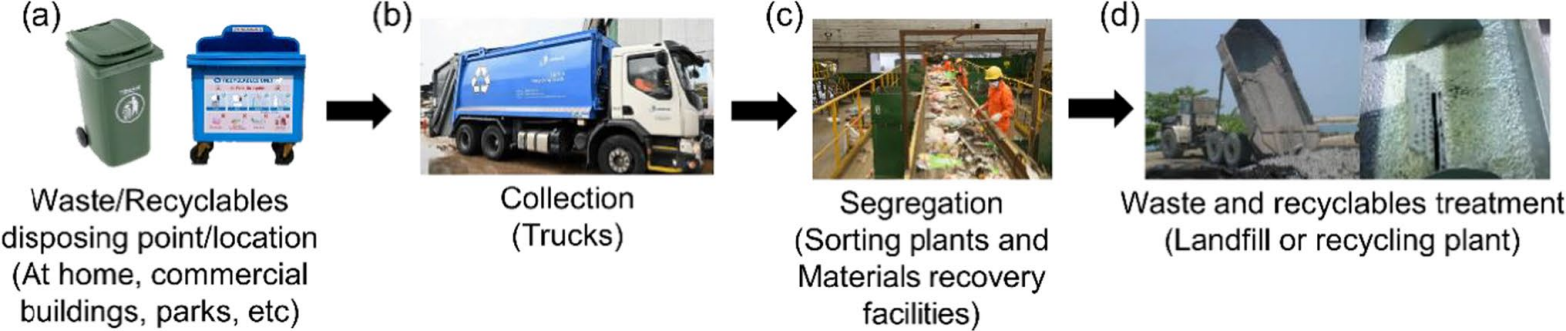
Typical processes of waste and recycling in Singapore and India (National Environment Agency 2019)

The process flow for managing plastic waste and recyclables (Fig. 3) in both Singapore and India includes (a) identifying the disposal point/locations; (b) collections by waste collectors; (c) segregation and (d) treatments to waste and recyclables.

Table 5 presents the comparison of waste and recycling infrastructure between Singapore and India. In Singapore, the NEA award licences to the public waste collector (PWCs) for providing waste collection and recycling services for domestic and trade premises and to the general waste collectors (GWCs) to provide services to commercial and industrial premises. Residents dispose the waste in the general waste bin or the collection point (i.e. rubbish chute and green bins around estates). The incinerable waste is collected by the waste collector and transported to the WTE incineration plants using a refuse collection vehicle (National Environment Agency 2020a). The WTE plant crushes the bulky waste with a high-capacity rotary crusher, followed by feeding the waste into the incinerator and burning it at temperatures of about 850 to 1000 °C (National Environment Agency 2020a). The heat produced during combustion generates steam in the boilers to drive the turbogenerators, which produced about 3% of the electricity needs in Singapore. The ash from the burnt waste has a volume of about 10% before incineration (National Environment Agency 2020a). The ashes are transported to the Tuas marine transfer station for disposal at the offshore Semakau landfill (National Environment Agency 2020a).

**Table 5:** Comparison of land area, economy and their waste and recycling infrastructure between Singapore (Ministry of the Environment and Water Resources 2019; National Environment Agency 2022b) and India (Ministry of Housing and Urban Affair 2019). Data related to country sizes and economy in 2020 were obtained from The World Bank (The World Bank 2020)

Singapore	India
Waste disposal point	
• General waste is disposed into the rubbish chute of HDB flats, and green-colour waste bin available around the estate, commercial buildings and parks • Recyclables are disposed into the blue-colour recycle bins located under each HDB flat and recycling bins around the estate, commercial buildings and parks • Mixed recyclables (paper, plastics, glass, metal) are disposed into the blue-colour bin and the recyclables are not separated by its respective material types at this point. Some commercial buildings, i.e. shopping centres, and parks initiate recyclables separation that offer recycling bins with instructions on the type of waste that can be binned	• Both general waste and recyclables are disposed, typically to be stored temporarily at home, yards and commercial buildings, prior to door-to-door collections • Waste is segregated into different streams, namely organic or biodegradable waste, dry waste (i.e. plastic, paper, metal and wood) and domestic waste (i.e. diapers, napkins, mosquito repellents and cleaning agents)
Collection	
• NEA appoints and licences the PWCs to provide refuse and recyclable services to serve domestic and trade premises • NEA licences the GWCs to serve the commercial and industrial premises • The waste collectors collect the refuse from the green bin and the recyclables from the blue bin by using trucks • The trucks transport the refuse from domestic and trade premises to the WTE plant. For the recyclables, they are transported to the MRF • For commercial and industrial premises, the GWCs collect the refuses and recyclables, followed by transporting them to the GWC’s facilities. Of note, GWCs can only handle the types of refuses and recyclables according to the licence that NEA has awarded to them	• Municipal authorities and Gram panchayat appoint waste collectors to collect the waste and recyclables by door-to-door collections from domestic premises, public places and storage in covered yard • The waste collection is heavily dependent on the informal sector, which includes waste collectors, trash/rag pickers, road sweepers, Dhalaos, intermediate dealers/junkyard owners and recyclers • Waste collected is transported to a segregation facility for sorting of waste and recyclables
Segregation	
• Waste collected from the waste disposal point is not segregated before sending off for waste treatment • Commercial and industrial waste collected by GWCs is generally segregated during collections. GWCs may require further segregations depending on the refuse types • Recyclables collected by PWCs or GWCs are usually sent to the collector’s MRF to segregate the recyclables into four different streams of materials (namely glass, paper, plastic and metal). Thereafter, the recyclables are consolidated and baled into large cubes (for paper and plastics only) • Non-recyclables found during segregation are removed and deemed as refuse	• Municipal authority and Gram panchayat to appoint registered facilities, and unorganized/informal sectors to segregate the waste and recyclables • The waste is segregated into incinerable waste, non-incinerable waste and recyclables • Subsequently, the segregated waste is transported to different facilities for waste and recyclables treatment
Waste and recyclables treatment	
• Incinerable waste is transported to the WTE incineration plants to reduce the waste into ashes. The heat generated from the superheated steam in the boiler drives the turbogenerators to produce electricity. After incineration, ferrous scrap metals are recovered and recycled, while the ashes are disposed at the offshore Semakau landfill • Recyclables are sent to several local recycling plants and mostly to the recycling plant located in neighbouring countries (Johor, Malaysia) • For plastic recyclables, they are further sorted into different plastic types. The plastic scraps are mechanically recycled by washing to remove contaminants, followed by shredding to become plastic flakes for remanufacturing. The flakes can be further process by pelletizing to form plastic pellets	• Incinerable waste is transported to plants for construction of bituminous road through hot mix plant, pyrolysis to convert waste to liquid fuel, co-processing in cement kilns as an alternate fuel and raw materials, and disposal through incineration • Non-incinerable waste is transported to plants for compositing (for organic waste), and grit making (for inorganic waste) • For plastic recyclables, they are further sorted into different plastic types. The plastic scraps are mechanically recycled by washing to remove contaminants, followed by shredding to become plastic flakes for remanufacturing. The flakes can be further process by pelletizing to form plastic pellets

In Singapore, NEA introduced a national recycling programme in 2001 that requires all PWCs to provide recycling bins and collection services to all estates, namely housing estate of the Housing Development Board (HDB), private landed properties and condominiums or private apartments, who opted into the public waste collection scheme (National Environment Agency 2022b). A standardized blue-colour recycling bin is provided at open area that is accessible to the residents and for fire safety reasons (National Environment Agency 2022b). The collectors (PWCs for domestic and GWCs for commercial and industrial) gather the recyclable materials (mixed recyclables) using a recycling truck (National Environment Agency 2019). The recyclables are transported to the material recovery facility (MRF) for sorting into different material streams (i.e. glass, paper, plastic and metal) (National Environment Agency 2019). During the process of sorting the recyclables, non-recyclables and contaminated materials will be disposed as refuse and sent to WTE plants for incineration (Ministry of the Environment and Water Resources 2019). The MRF consolidates the recyclables into bales and transports them to the recycling plants (National Environment Agency 2019). For plastic recycling, the recycling plant sorts the recyclables to their polymer type (if possible, based on resin identification codes). Thereafter, mechanical recycling is conducted by shredding the plastics into flakes and washing the flakes to remove contaminants (National Environment Agency 2019). The plastic flakes can be further processed by pelletizing them into granules. The plastic flakes or granules can be served as feedstock for remanufacturing (Jen Teo 2018; National Environment Agency 2019).

India embraces a similar plastic waste and recycling management strategy. In particular, the types of plastic recycling processes and facilities implemented in India are quite similar to those in Singapore. Waste and recycling management in India are conducted at the municipal level (Ministry of Housing and Urban Affair 2019). The municipal authority or gram panchayat appoints local waste collectors to collect waste and recyclables from domestic and public places and segregate them into incinerable waste, non-incinerable waste and recyclables (Ministry of Environment 2016a). Unlike Singapore (a developed country) which engages licenced waste collectors, India (a developing country) depends heavily on the informal sector, namely waste collectors, trash/rag pickers, road sweepers, Dhalaos, intermediate dealers/junkyard owners and recyclers which contributes to a relatively high plastic collection rate of 60% (Ministry of Housing and Urban Affair 2019). While the formal recyclers (formal sector) are generally part of the waste management chain to manage the bulk plastic waste that are collected by the informal sector (Ren et al. 2021). Furthermore, uncollected plastic wastes are often openly dumped due to a lack of properly engineered landfills and also leads to poor practices where open burning of waste is conducted to reduce the waste volume (Ren et al. 2021). Over the years, India has been utilizing the non-recyclables and co-processed it for road construction and in cement kiln, including pyrolysis to obtain fuel from waste (Ministry of Housing and Urban Affair 2019). Currently, Singapore is still researching on plastic recycling methods using gasification and pyrolysis to obtain useful products. More importantly, in land-scarce Singapore, only some recyclables are recycled locally, and most of the recycling processes are conducted in neighbouring countries (Johor, Malaysia) (Kerdlap et al. 2020, 2021), while India is able to house all the recycling processes and facilities locally (Ministry of Housing and Urban Affair 2019).

### Recycling statistics

**Fig. 4:**
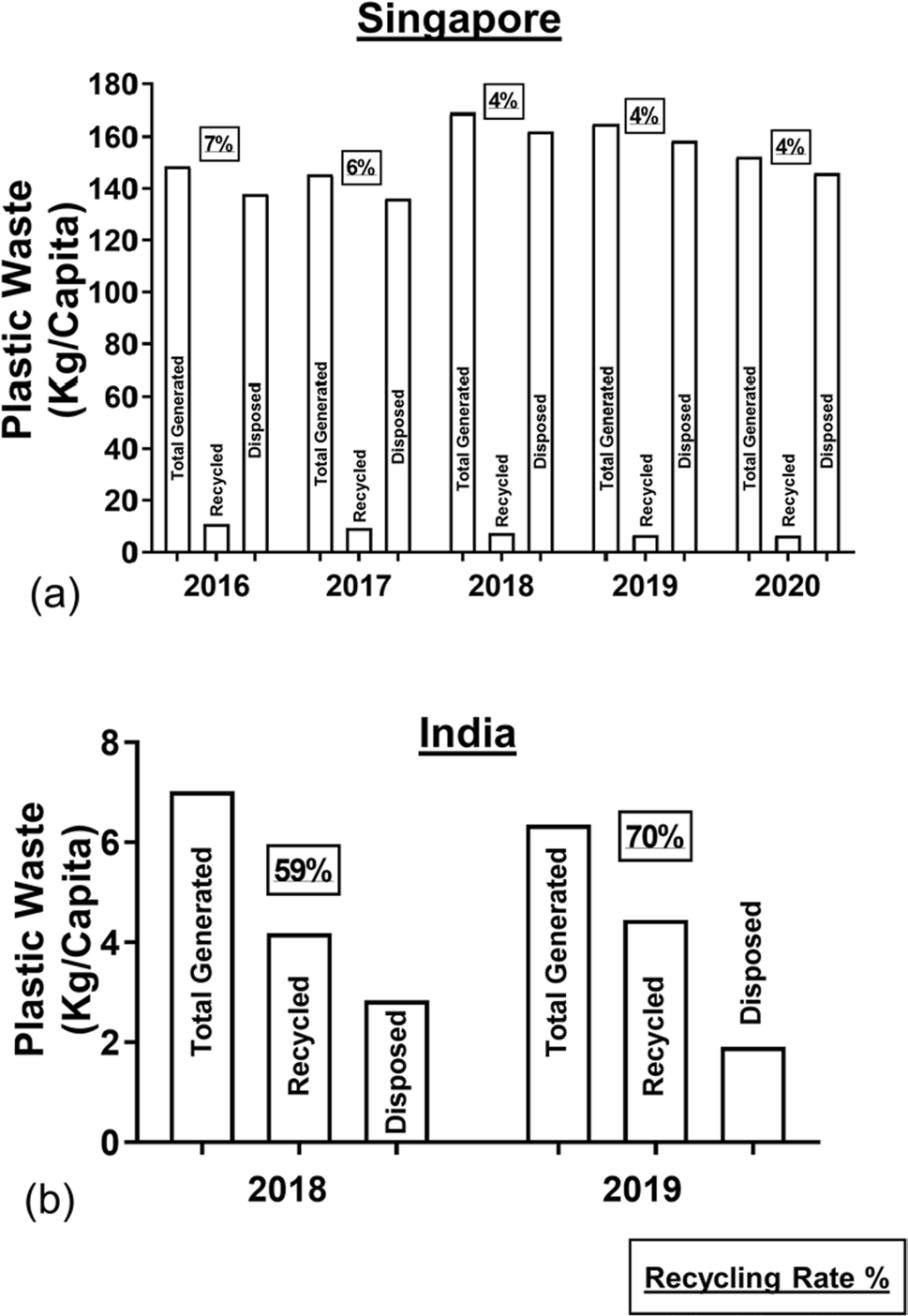
Bar charts of plastic waste generated, disposed and recycled in **a** Singapore (2016–2020) (National Environment Agency 2021d) and **b** India (2018–2019) (Ministry of Housing and Urban Affair 2019)

NEA (Singapore) reported that a total of about 868 thousand tonnes (152 kg per capita) of plastic waste were generated, whereas about 36 thousand tonnes (6 kg per capita) were recycled in 2020 (see Fig. 4a) (National Environment Agency 2021a). In 2018, a report from Singapore Environment Council (SEC) revealed that packaging waste contributed to a huge portion of the total plastic waste in Singapore (Dahlbo et al. 2018). The types of packaging waste consist of about 467 million PET items (i.e. bottles), 473 million PP items (i.e. takeaway containers) and 820 million PE items (i.e. plastic bags) (Singapore Environment Council 2018). It was observed that the overall plastic recycling rate had decreased from 7% in 2016 to 4% in 2020, and the residual waste was sent to WTE incineration plants, followed by disposing off in offshore Semakau landfill (Elangovan 2021; National Environment Agency 2021d).

The decreasing recycling rate was mainly attributed to contamination of recyclables due to incorrect disposal of non-recyclables into the recycle bins (Mahmud 2018). Another factor that resulted in the decrease of the recycling rate was attributed to the decrease in overseas demand for recyclable materials. For instance, the ban imposed by China for importing recyclable materials, and restrictions on recyclable materials imposed by Malaysia and Thailand have impacted the economic value of the recyclable materials (Rapoza 2020). The recyclable materials generate low-profit margins in considering the cost to collect at source (Mohan and Min 2020).

In India, the CPCB reported that a total of 8.6 million tonnes (6 kg per capita) of plastic waste were generated, 6 million tonnes (4 kg per capita) of plastic waste were recycled, and the remaining 2.6 million tonnes (2 kg per capita) of plastic waste were confined to landfill or left squandered in 2019 (Central Pollution Control Board 2019; Ministry of Housing and Urban Affair 2019). The total plastic waste generation has reduced by 8% as compared to 2018. Concomitantly, it was observed that the overall recycling rate has increased from 59% in 2018 to 70% in 2019 (Central Pollution Control Board 2018, 2019). However, plastic waste leakage during the recycling process attributable to the dearth of formal recycling channels, standard operating procedures and rudimental strategies steer is a major challenge facing by the recycling industry (Kapur-Bakshi et al. 2021). The formal recycling sector is predominantly constricted to cleanse and sequester pre-consumer waste by regions in the country. Encompassing those in the western states of Maharashtra and Gujarat benefits from the rugged transport and recycling infrastructure and grid connectivity (Kapur-Bakshi et al. 2021). Notably, high transportation costs were incurred as the pre- and post-consumer recyclables from distant states of Southern or Eastern India were also recycled in the western states of India (Ministry of Environment 2016a). Poor infrastructure and road connectivity in regions, especially North-East India, pose a major challenge due to the long-distance transportation of waste in India. Furthermore, the available recycling infrastructure could only process up to 50–60% of the total recyclables, as the remaining recyclables were contaminated and rejected. In addition, the ban on the import of plastic waste in India has relieved stresses on the recycling sector in managing the high volume of plastic recyclable (Kapur-Bakshi et al. 2021).

CPCB has summarized the challenges in managing waste in India as follows: (1) inadequate data submission from the state; (2) data submitted by the responsible organization does not comply with the waste reporting standard as published under the waste management rules; (3) waste were not fully accounted owing to incomplete data submission from some states and (4) rampant illegal dumping and failing to comply with the waste management rules (Central Pollution Control Board 2018, 2019).

## Plastic waste conversion technologies for PET

In this section, three plastic waste treatment technologies for PET, namely mechanical recycling, pyrolysis and incineration were discussed. The technologies have been summarized and presented in Table 1. As the name suggests, mechanical recycling of plastics such as PET processes plastic products by mechanical means, such as grinding, washing, separating and drying, to recover the PET with minimal alteration to the material. Pyrolysis is a process which subjects plastic waste to thermal degradation (in the absence of oxygen) to recover fuel or monomer. Thermal degradation reduces long-chain polymer molecules to smaller, less complex molecules under the effect of pressure and heat in the absence of oxygen (Damayanti and Wu 2021). Incineration is a terminator process of eliminating plastic waste by the combustion of plastic waste to ashes (thus reducing the original physical volume of the waste by 85–90%) before disposal takes place; the process in turn produces heat for powering devices.

Moreover, with the help of mechanical recycling, the recovered PET could be used for manufacturing products such as clean energy devices (TENG) (A potential energy harvesting device made from recycled PET). After going through several cycles of recycling, degradation in the physical and chemical properties of PET could reduce the economic value (Retrofitting PET waste treatment towards a sustainable circular economy). Eventually, the PET waste could be treated through the pyrolysis or incineration process. The key strength and weaknesses of the respective technologies have been highlighted. A major concern with mechanical recycling is the presence of different contaminants including heat stabilizers, additives, plasticizers, pigments, flame retardants and concoction of different polymer types affect the quality of secondary plastics inferring low economic and technical values. The pyrolysis technology currently requires high carbon and energy inputs which limits its implementation on industrial scales. The incineration technology is limited by large greenhouse gas emission and landfills are needed to accommodate the ashes (National Environment Agency 2020b).

### Mechanical recycling

To date, the mechanical processing of plastics is the most traditional and economical technique employed for recycling post-consumer plastic waste (Geyer et al. 2017). A large proportion of the recovered post-consumer PET bottles are recycled through mechanical recycling in India and Singapore (Central Institute of Plastics Engineering & Technology 2008; Khoo 2019). However, the recycled materials could only be employed for applications that are not intended for exigent performance.

Mechanical recycling could be operated in two modes, namely primary and secondary. The primary recycling mode, otherwise known as ‘closed loop recycling’, refers to reprocessing of plastics to manufacture products utilized for the same purpose as the pristine materials. The secondary recycling mode refers to the utilization of reclaimed materials as a source of new production (National Environment Agency 2019). These modes of recycling involve sorting, shredding, washing and pelletizing (or extrusion to form pellets) of the material as described in the “Plastic waste management infrastructure” section.

The polymer materials are limited by the number of reprocessing cycles, due to the shortened polymeric chains after chain scission amortize polymer viscosity and elasticity and embrittles the polymer (Hopewell et al. 2009). The presence of contaminants and additives complicates the processes and affects the amount of recovered materials (Hopewell et al. 2009). Residues of PVC, PLA and PVA in PET can leach acids and stimulate hydrolysis or acidolysis of PET during extrusion. Contaminated plastic trash demands rigorous cleaning prior to recycling, which, even with state-of-art technology, involves the utilization of about 2 to 3 m^3^ of water per tonnes of materials (Damayanti and Wu 2021). Furthermore, cross-linking during chain extension (due to an increase of molecular weight) adversely affects the recyclate quality and risk damaging the equipment used for the recycling processes. The degradation of PET chains is prompted by carbon to hydrogen transfers or attacks by free radicals. As a result of the heat and the stress from the extruder, the reaction of macroradicals with oxygen occurs, resulting in the formation of peroxy-based radicals. Emanating macroradical chains as a result of radical hydrogen abstraction exacerbates the concentration of carboxylic acid end groups and causes thermo-oxidation. Henceforth, the recyclables are more prone to degradation after each recycling cycle due to the degradation of the polymer chain (Hopewell et al. 2009).

The microstructure of the PET could take on the following forms: rigid amorphous, mobile amorphous and crystalline. The mobile amorphous fraction is more prone to attack, releasing short chains to fold into intercrystalline domains nucleate crystallization (Vollmer et al. 2020). Thickening and rearrangement of the crystalline domains can occur, resulting in new crystalline domains with smaller average sizes formed with increased recycling, causing increased embrittlement and stiffness. Irrevocable impairment of polymeric chains as a result from mechanical recycling leads to the final disposal in landfills (Vollmer et al. 2020).

### Pyrolysis

**Fig. 5:**
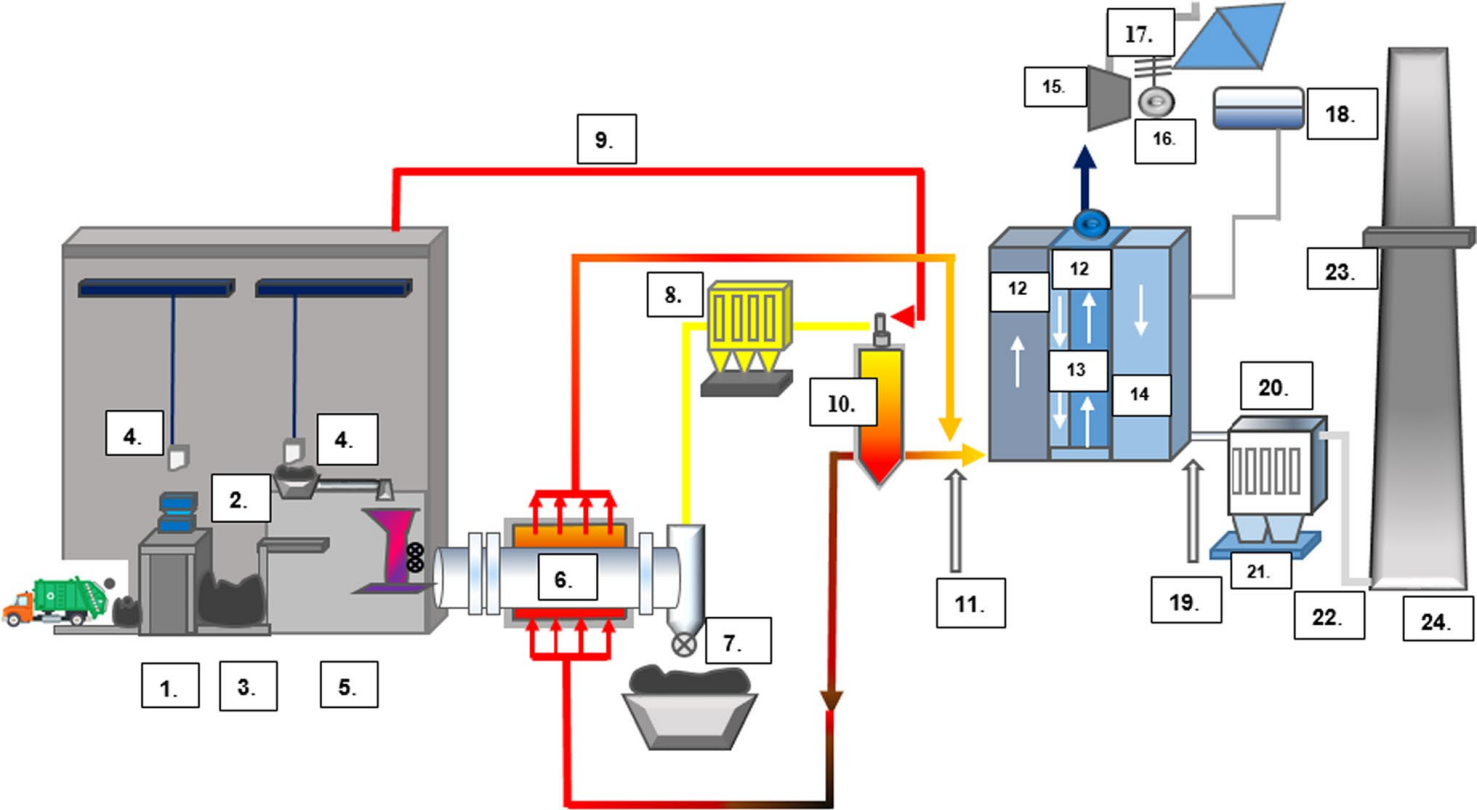
Schematic of a general pyrolysis plant: (1.) coarse refuse bunker, (2.) rotary shares, (3.) fine refuse bunker, (4.) overhead crane, (5.) feeding system, (6.) pyrolysis kiln, (7.) discharging system, (8.) hot gas filter, (9.) combustion air fan, (10.) combustion chamber, (11.) selective non-catalytic reduction, (12.) evaporator, (13.) superheater, (14.) economizer, (15.) turbine, (16.) generator, (17.) condenser, (18.) feedwater tank, (19.) additive metering hopper, (20.) fibrous filter, (21.) filter dust discharging, (22.) induced draught ventilator, (23.) the emission monitoring system, (24.) stack

A schematic of a typical pyrolysis plant is presented in Fig. 5. The pyrolysis process has been used to recuperate energy and fuels. The pyrolysis process can form (1) gases, comprising light-weight hydrocarbons, hydrogen, CO and CO_2_; (2) oil/wax mixture, encompassing a mixture of aliphatic and aromatic compounds and (3) char, a solid residue which is derived after processing. At lower temperatures, oil and waxes are formed; at higher temperatures, pyrolysis leads to monomer recovery in larger quantities.

Degradation can occur in the following ways under process conditions: (a) depolymerization into monomer; (b) randomly breaking the polymer chain into smaller fragments; (c) removal of side groups or reactive substitutes; (d) cross-linking in case of thermosetting polymers during heating. Desired products could be obtained by varying the process parameters (i.e. temperature, catalysts, residence time, pressure, type of fluidizing gas and its rate, type of reactors) (Dogu et al. 2021).

Pyrolysis has been investigated as an appealing substitute for incineration for waste disposal. In contrast to the conventional incineration plant operated in the capacity of kilotonnes per day, the scale of the pyrolysis plant is more versatile, and the output of pyrolysis can be consolidated with other downstream technologies for product upgradation (Peng et al. 2022; Yansaneh and Zein 2022). The liquid oil yielded can be utilized in several applications, including boilers, furnaces, diesel engines, and turbines, without further treatment (Rajendran et al. 2019).

Compared to mechanical recycling, the pyrolysis technology is in the early stage of development and a majority of the technologies were in pilot scale in both countries (Khoo and Tan 2010; Kapur-Bakshi et al. 2021). High production cost, energy inputs and release of harmful chemicals, for instance, biphenyls and polycyclic compounds could further limit its application (Roosen et al. 2020). Several small-scale pyrolysis systems have been implemented in India (Dutta and Bhaskar 2017) and Singapore (National Environment Agency 2020c), but more research are needed namely on the design, environmental impact of the incineration before full-scale commercialization in both countries could be realized.

### Incineration

**Fig. 6:**
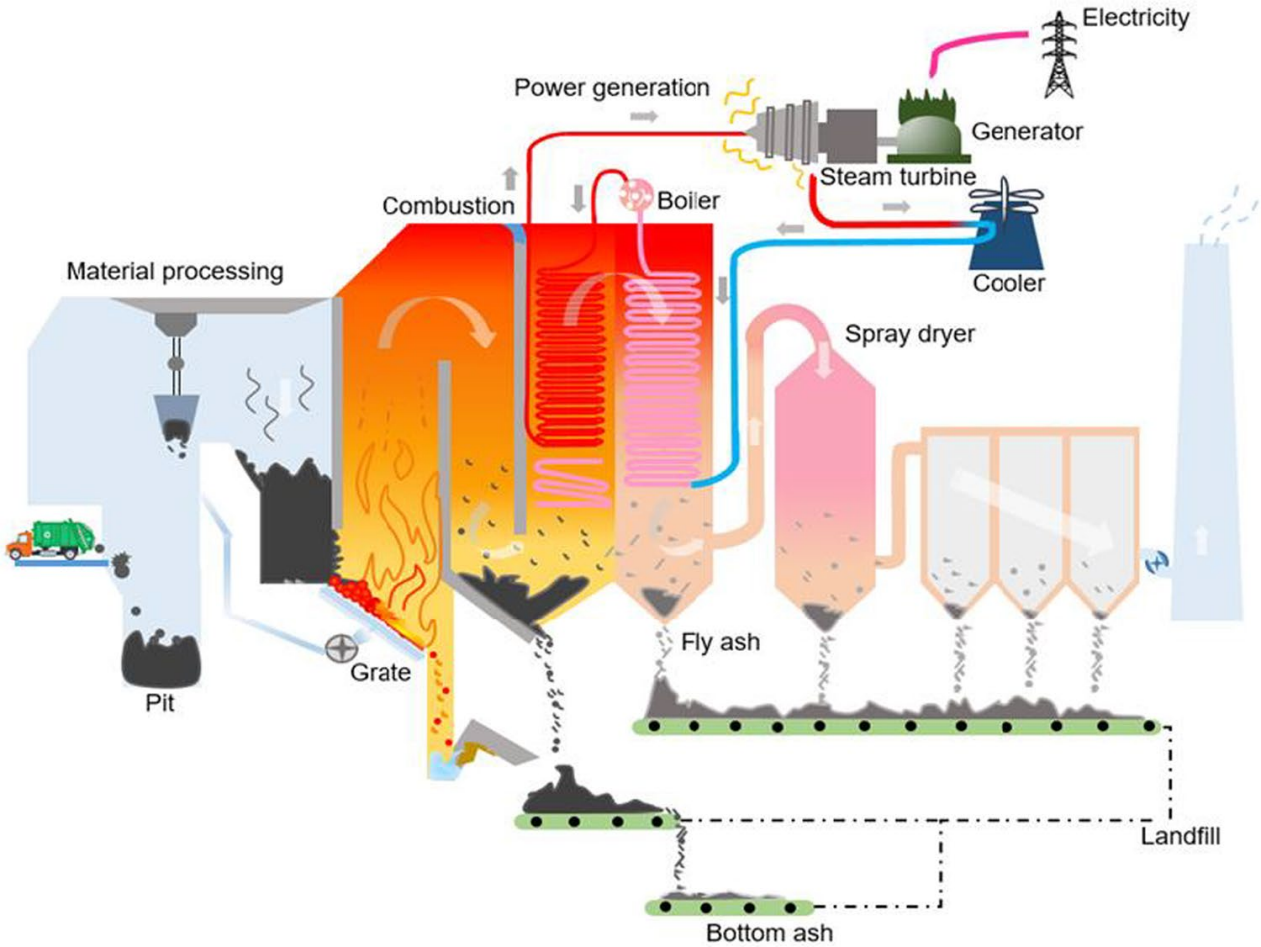
Schematic of an incineration plant

In Singapore, incineration (see Fig. 6) is a common method to process waste material to derive energy while minimizing the volume of waste sent to the landfill. It was highlighted that about 48% of the 5.88 million tonnes of plastics and non-plastics generated in Singapore were incinerated in 2020 (National Environment Agency 2021a). However, India restricts plastic waste incineration to multi-layered plastics and non-recyclables (Ministry of Environment 2016b).

At a typical incinerator (Fig. 6), plastics are burnt to ashes to generate heat which is used to boil water to release steam to turn turbine blades and this in turn generates electricity for the local grid. Concomitantly, this is accompanied by the release of greenhouse gases and toxic pollutants like acid gases, heavy metals and dioxins (Sharma et al. 2021). The utilization of recovered energy (recovered in the form of heat after incineration of waste) varies noticeably which depend on the energy recovery method such as the combination of power and heat, generation of electricity and solid refuse fuel for cement kilns or blast furnaces (Yang et al. 2021). Incineration of plastic waste requires a large amount of energy and the energy generated by a mass burning of plastics were substantially less than the energy conserved by recycling (Rahimi and Garciá 2017). A study has reported that the energy recovered from the incineration of plastic scraps was about 36,000 kJ/kg, whereas the processing of plastic scraps through mechanical recycling conserved approximately 60,000 to 90,000 kJ/kg (Rahimi and Garciá 2017). Thus, mechanical recycling of plastic scraps eventually conserves more energy than incineration can generate (Gradus et al. 2017). Furthermore, there are concerns about the environmental impacts of the pollutants. The by-products such as CO_2_, acidic gases (oxides of sulphur), persistent organic compounds (dioxins and furans), heavy metals and particulate matters are highly hazardous which can result in global warming and several health problems including respiratory symptoms, decreased lung function and high cancer risk (Zhang et al. 2021).

## Retrofitting PET waste treatment towards a sustainable circular economy

In this section, the feasibility considerations for a circular economy approach for the PET have been discussed. To begin, the economic value was compared to the performance of the PET recyclates after consecutive mechanical recycling. Thereafter, when it is no longer economically viable to recycle the PET waste (i.e. when it is no longer feasible to keep the PET in circulation), the existing waste conversion technologies were explored as an end-of-life strategy for eliminating the PET waste and its impact on the environment.

### Economic value of PET recyclate materials

Thus, plastic recycling to recover PET can be achieved through mechanical recycling. Mechanical recycling is a common method used in many countries to process the plastic scraps to secondary raw materials (see “Mechanical recycling” section) (Gu et al. 2017; Khoo 2019; Ren et al. 2021; Sharma et al. 2021). Often, the mechanical properties of the recycled plastics were inferior as compared to the virgin plastics. Therefore, to mitigate the potential loss of property, as a foundation for future efforts, an economic analysis to establish the future closed-loop material usage of PET materials is presented with regard to the environmental impact and externalities. Existing life cycle analysis studies on the existing plastic conversion technologies were analysed to investigate the environmental benefit of a technology against the existing technologies in use.

In this section, a model was employed to evaluate the material economic value for the sustainability and impact of material flow in a closed-loop circular economy (Hagnell and Åkermo 2019). Herein, the model established the economic value of the plastic scraps after consecutive mechanical recycling for evaluating the sustainability of using recycled plastics in manufacturing (National Environment Agency 2021b). The economic value of the recyclable materials (${RV}_{i}$) may be estimated using a recyclate value model (RVM) developed by Hagnell and a co-worker (Hagnell and Åkermo 2019). The RVM considers the mechanical performance of the recycled materials after each recycling step/cycle ($i$). The RVM can be expressed as Eq. (1),

1 $$\begin{array}{c}{RV}_{i}=f\left({RV}_{i-1}\right)=m{r}_{p}{RV}_{i-1}-P\\ { RV}_{i}\ge B\end{array}$$

where ${RV}_{i}$ is the recyclate material value after recycling. ${RV}_{i-1}$ is the recyclate material value before the current recycling step/cycle. $m$ is the retained mechanical performance factor (0 ≤ *m* < 1) of the recycled materials to account for the material degradation effects (such as thermal, chemical and loss of materials) during the recycling process. ${r}_{p}$ is the percentage of reclaimed recyclate yield that accounts for the amount of contaminants after recycling. $P$ represents the recycling process cost. ${RV}_{0}$ is the economic value of the materials when it is virgin (not recycled before). $B$ is the final value after the recycling process.

Table 6 shows the data used to derive the ${RV}_{i}$ of PET plastic. The estimated ${RV}_{0}$ of the virgin PET for South-East Asia (Singapore) were obtained from S&P Global Platts, while the ${RV}_{i-1}$ of the recycled PET flakes were obtained from a company (2 Lians Pte Ltd) located in Singapore. The estimated ${RV}_{0}$ of the virgin PET in South Asia (India) were obtained from a company in India (Rishav Polyplast Pte Ltd), while for the estimated ${RV}_{i-1}$ for the recycled PET flakes were obtained from Jebruna International Pte Ltd. Notably, the ${RV}_{i-1}$ of the PET materials were estimated from several companies in March 2021. Furthermore, a 30% decrease in ${RV}_{i-1}$ of PET following each recycling step was conjectured. The dataset ($m$) for the mechanical performance factor (namely tensile strain properties, Charpy impact properties, the viscosity of PET resin during extrusion and degree of crystallinity) of PET plastic were obtained from several published articles (La Mantia and Vinci 1994; Del Mar Castro López et al. 2014; Schyns and Shaver 2021). With regard to the ${r}_{p}$, the virgin state of PET plastic was assumed to be devoid of any contaminations and a proliferation of about 3% was projected with each consecutive recycling step. With increasing recycling step, it was assumed that there were about 3% contaminants (which may be due to a mixture of different colours of PET recyclables). For *P*, a fixed value was assumed to avoid complexity, but in reality, the recycling costs may differ in different countries depending on the operational capacity of facilities and on the recycling method and material types.

**Table 6:** Predictions of the recyclate value of PET plastic

Country	No. of times recycled?	Estimated economic value, *RV*_*i-1*_^#^ (USD/mt)	Retained mechanical performance factor (m) (0 ≤ *m* < 1)				Reclaimed recyclate (*r*_*p*_) ^§^	Recycling cost (*P*) ^‡^
			Elongation at break (*ε*_*B*_)	Impact strength (*α*_*cU*_)	Viscosity (*η*)	Degree of crystallinity (*D*)	(0 ≤ *r*_*p*_ < 1)	
Singapore	0	1341	1	1	1	1	1	50
	1	610	0.833	0.526	0.97	0.97	0.97	
	2	427	0.136	0.185	0.94	0.94	0.94	
	3	299	0.062	0.119	0.91	0.91	0.91	
	4	209	0.038	0.044	0.88	0.88	0.88	
	5	146	0.018	0.044	0.85	0.85	0.85	
India	0	1090	1	1	1	1	1	50
	1	680	0.833	0.526	0.97	0.97	0.97	
	2	476	0.136	0.185	0.94	0.94	0.94	
	3	333	0.062	0.119	0.91	0.91	0.91	
	4	233	0.038	0.044	0.88	0.88	0.88	
	5	163	0.018	0.044	0.85	0.85	0.85	

#Material value dropped by 30% from the previous recycling step

**§**Materials purity decreases by 0.03% from the previous recycling step

**‡**No change in the process costs per recycling step

**Fig. 7:**
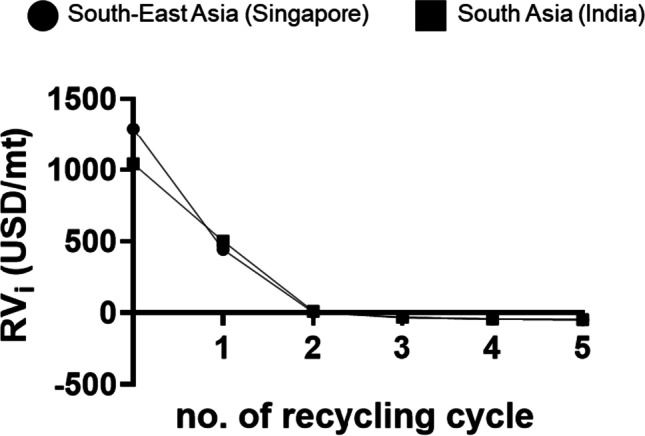
Estimated recyclate value (${RV}_{i}$) of each recycling step or cycle (up to 5 cycles) of PET materials in South-East Asia (Singapore) and South Asia (India)

Figure 7 shows the line chart of the estimated $${RV}_{i}$$ of each recycling cycle (up to 5 cycles) for PET materials. It was observed that the $${RV}_{0}$$ (sale value) for South-East Asia (Singapore) was higher than South Asia (India) when the PET materials were in the virgin state. After the first recycling cycle ($$i$$ = 1), the estimated resale value ($${RV}_{1}$$) of the recycled PET material decreases about 65% (Singapore) and 52% (India) as compared to the virgin ($$i$$ = 0) PET materials. When the recycled PET materials ($$i$$ = 1) were introduced into remanufacturing and thereafter reached the end of the useful life of the products, the PET materials were considered for recycling again ($$i$$ = 2) to reuse and keep the PET materials within the closed-loop economy. The resale value ($${RV}_{2}$$) of the PET material reduces to US$0 per metric tonnes. Furthermore, recycling results in negative $${RV}_{i}$$ values, suggesting that the costs of mechanical recycling processes were more expensive than the resale value of the recycled PET materials ($${RV}_{i}<B$$) after the second cycle.

**Fig. 8:**
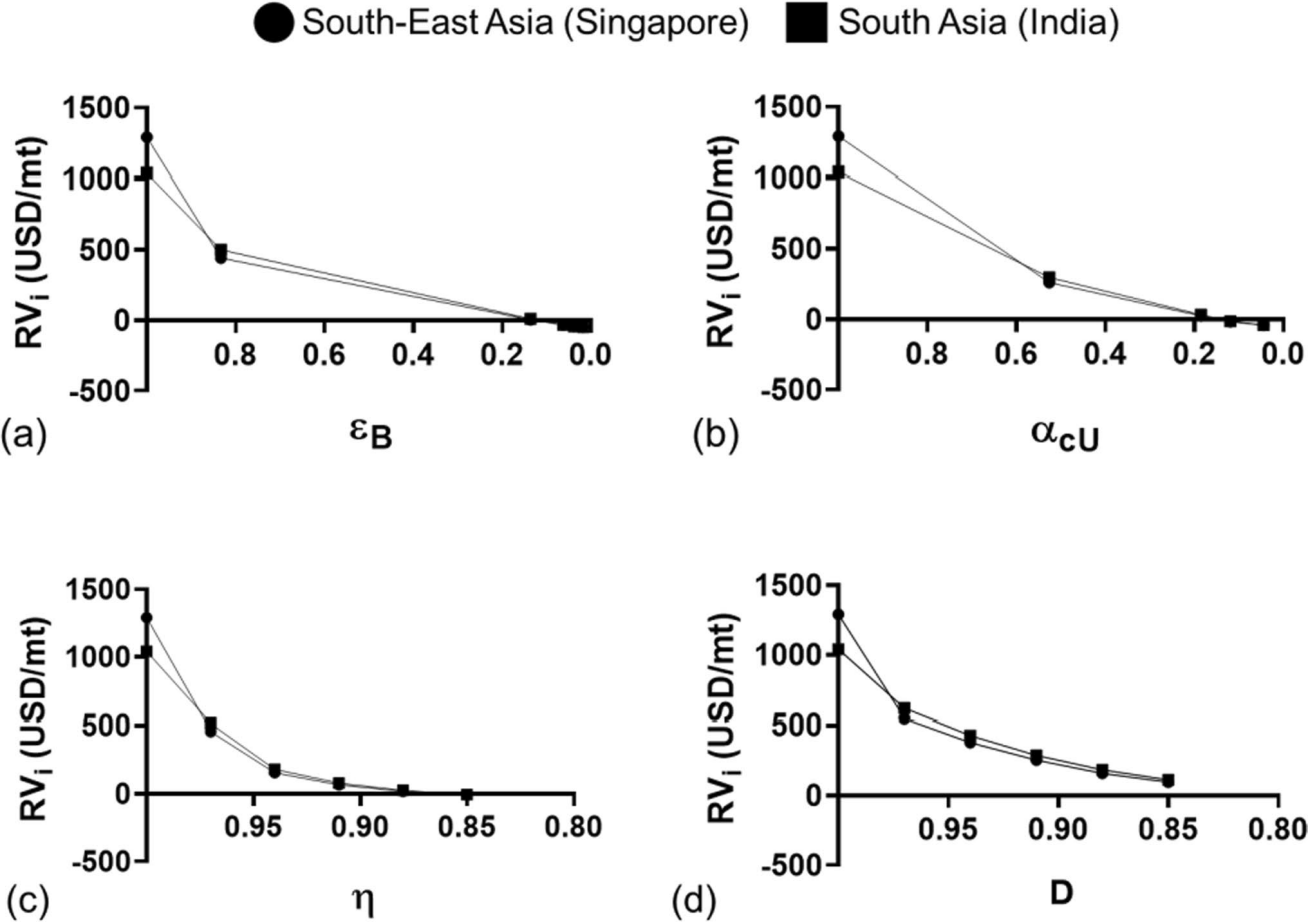
Estimated recyclate value (${RV}_{i}$) to the mechanical performance factor (Del Mar Castro López et al. 2014) of virgin PET and recycled PET in South-East Asia (Singapore) and South Asia (India). **a** ${RV}_{i}$ versus elongation at break, ε_B_. **b** ${RV}_{i}$ versus Charpy impact strength, α_cU_. **c** ${RV}_{i}$ versus viscosity of PET resin during extrusion, η. **d** ${RV}_{i}$ versus degree of crystallinity, D

Figure 8 shows the line chart of $${RV}_{i}$$ with respect to the mechanical performance factor, namely (a) rupture strain, *ε*_*B*_, (b) Charpy impact strength, *α*_*cU*_, (c) viscosity of PET resins during extrusion, *η*, and (d) degree of crystallinity, *D*, of the virgin PET and recycled PET in South-East Asia (Singapore) and South Asia (India). In general, the $${RV}_{i}$$ decreases non-linearly along with the decreasing mechanical performance of the recycled PET, regardless of region and country. The estimated value ($${RV}_{i}$$) of the recycled PET materials depreciate close to no value (USD$0) when the mechanical performance factor of *ε*_*B*_ and *α*_*cU*_ decreases to a magnitude of smaller than 0.2, and the *η* and *D* decrease to a magnitude of smaller than 0.88 (for more details on the economic value data of recycled PET plastics, refer to Supplementary Information, Section 1.)

The analysis concluded that the economic value of PET depreciates after mechanical recycling as compared to the economic value of virgin PET materials. The economic values depreciate to zero value with the decreasing mechanical performance of PET after recycling for several cycles. Revealing that the recycled PET materials may be only suitable for remanufacturing for certain applications where the product design does not require high mechanical performance (i.e. micro-mesh fabric used for wastewater treatment and TENG for energy harvesting). Further estimation of $${RV}_{i}$$ (when $$i$$ = 3, 4 and 5) revealed that it is not economical to recycle the PET materials for more than two cycles due to the costs incurred for the mechanical recycling processes (where $${RV}_{i}$$ in the negative region shown in Fig. 7) and the reduction of mechanical performance after multiple recycling cycles (Fig. 8). Of note, when the materials were dumped in the landfill, the economic value of the materials would be USD$0 per metric tonnes (Hagnell and Åkermo 2019).

### Circular economy

New clean energy technologies to reduce emission such as TENG rely on raw materials (namely PET) to build the device. The manufacturing process would increase pressure on the supply of PET materials. How would this also increase pressure on our natural resources will depend on the manufacturing efficiency and keeping the PET in circulation for as long as possible?

**Fig. 9:**
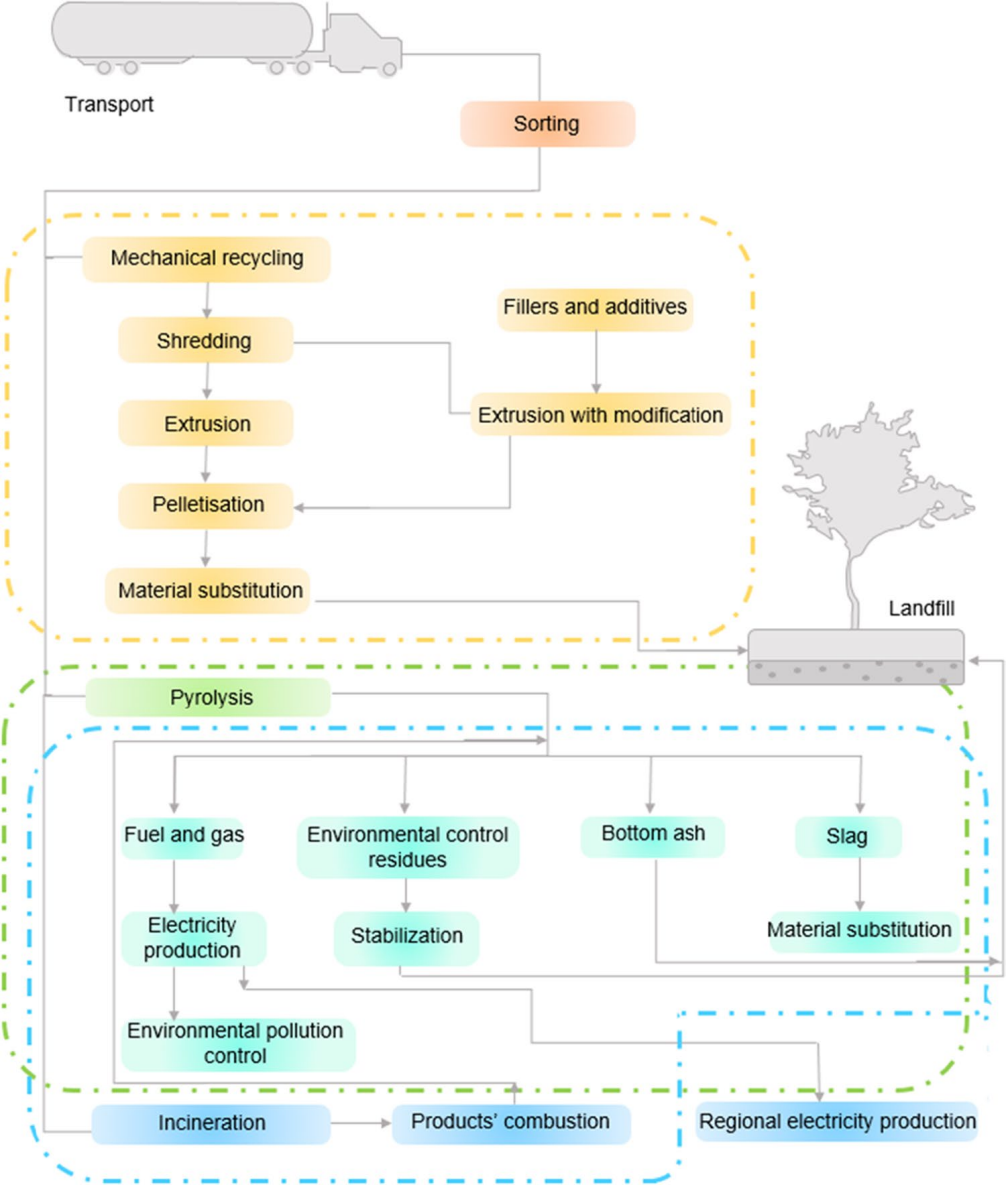
System boundaries for end-of-life assessment of PET. The types of systems include mechanical recycling, pyrolysis and incineration

Figure 9 illustrates the processes of system boundaries of end-of-life assessment of PET including the process of waste conversion technologies such as mechanical recycling, pyrolysis and incineration. Mechanical recycling of post-consumer PET waste encompasses a series of events, including sorting, cleaning, drying, size reduction and reprocessing. Treatment techniques such as bottle washing, grinding and flake washing could result in a material loss (8%); a further material loss could occur through the following processing stages: melt filtration (1%), air classifier (7%), material conveying (2%) (Sherwood 2020). Typically, the mechanical recycling processing temperature ranges from 280 to 320 °C (Chaudhari et al. 2021). In India and Singapore, mechanically recycled PET materials were utilized to make polyester fibres. In India, it was predicted that about 95% of recycled PET was utilized to make polyester fibre materials (NCL Innovations, CSIR-NCL 2018). In 2018, a national life-cycle analysis (LCA) report published by CPCB on packaging plastics projected that a total energy of 31651.2 MJ was consumed for processing 1 tonne of PET bottles by mechanical recycling. The report also published the amount of water consumed (about 7529 L) and the emission of contaminants that were noxious to the environment such as carbon monoxide, nitrogen oxide, sulphur oxide and several dust particles. About 1001 kg of carbon dioxide emission (which contributes to the greenhouse effect) was predicted to be released (Meys et al. 2020). For Singapore, a study has assessed the life cycle of PET waste (Khoo 2019). A significantly lower greenhouse gas emission attributable to the employment of hydroelectric energy for powering the mechanical recycling processes was observed (Khoo 2019). It was concluded that mechanical recycling was not widely used in Singapore; instead, the WTE plants were used widely for PET disposal (Khoo 2019, Grace Yeoh 2021).

The three principles of a circular economy are concerned with design for (i) the elimination of waste and pollution, (ii) circular handling of products and materials in use and (iii) the regeneration of natural systems (Ministry of Sustainablility and Environment 2021; Velenturf and Purnell 2021). A closely associated notion with circular economy is that sustainable development with the environmental and economic dimensions of sustainability creates significant coherence with the principles of circular economy (Priyadarshini and Abhilash 2020). Evaluation of the environmental impact of the entire life cycle of plastics (from design to disposal) have identified opportunities for innovative solutions and systemic changes to address the sustainable development challenges (Huysman et al. 2017; Cordier and Uehara 2019; Foschi et al. 2020; Cordier et al. 2021).

A priority for the management of waste in order to guarantee the selection of the most environmentally sound option is to establish the resource hierarchy. The actions associated with this hierarch were highlighted by the R-ladder which was used to evaluate the end-of-life options (Keijer et al. 2019). The R-ladder refers to reject, reduce, reuse, redistribute, repair, refurbish, repurpose, remanufacture, recycle, recover and return. The least preferred option in this ladder of circularity is the ‘landfilling of waste’ or ‘incineration of materials as waste’. It is important to note that even though incineration as a disposal method generates electricity and heat, this should not be generally recommended unless there are no viable recycling options (Hahladakis et al. 2020). While prevention of waste (by avoiding or preventing the use of plastics) is the most desirable option, an optimal process design for the efficient separation, purification, recycling and reuse of waste products is the best option for waste management in case waste generation is not avoidable. With regard to PET, the processes to recycle PET waste should not result in increased CO_2_ emission relative to new virgin PET materials.

### End of life assessment of PET

When the economic value of PET recyclate diminute after multiple recycling as shown in the “Economic value of PET recyclate materials” section, what could be done with the PET waste if recycling is no longer viable?

The pyrolysis method is an environmentally friendly (conserving fossil resources) method as compared to the incineration method as it could aid in curbing environmental pollution and reduction of global warming (Czajczyńska et al. 2017). Compared to municipal solid waste incinerators, pyrolysis plants require lower process temperature and result in lower air pollutant emission (Czajczyńska et al. 2017). In comparison to waste processing by cement kilns, monomer production by pyrolysis give rise to negative environmental potentials for LDPE and HDPE (high calorific HDPE and LDPE must be compensated by lignite in cement kilns resulting in higher emissions), a small positive for polypropylene (the increase in avoided global warming impacts in cement kilns is slightly less than for HDPE and LDPE), and positive environmental potentials for polystyrene and PET (global warming impacts were avoided from conventional production of styrene and ethylene) (Meys et al. 2020). In comparison to mechanical recycling, pyrolysis could recover gases and fluids, and refinery feedstock to avoid small-scale global warming (Meys et al. 2020). The current scenario suggests that the pyrolysis plants presently employed in the place of municipal waste incinerators reduce terrestrial acidification and marine/freshwater eutrophication if refinery feedstocks or fuels are produced. On the other hand, all the processes present many opportunities to reduce terrestrial acidification if cement kilns were utilized for energy recovery (Vollmer et al. 2020).

Incineration of PET waste could contribute negatively to the waste pathway and waste management, as such the practice causes plastic residuals (i.e. filter ashes, bottom ashes and sludge after incineration) to remain trapped in the ecosystem which ultimately requires landfill disposal. Decomposition in landfill results in the production of bad odours and landfill gas that is toxic to humans and could contribute to acidification and global warming (Sharma and Chandel 2017). The ashes comprised of toxic pollutants, such as heavy metals, which could leak into the marine environment in the form of leachate through groundwater. In Singapore, the main contribution to the greenhouse gas comes from the WTE plants; a large proportion of the residual of the waste or ashes were sent to the off-shore Semakau landfill (Kerdlap et al. 2021). In India, about 8% of plastic waste was sent to cement kilns for incineration. Incinerating the plastic waste in cement kilns contributed to 38% of total climate change by pollution from the gases and the ashes (Ren et al. 2021). While sanitary landfills (installed with walls that isolate the trash from the environment to protect it from contamination by leachate, emitted landfill gas, etc.) have been engineered and established in both countries, it has been predicted that the effectiveness of these measures would degrade with time (Ren et al. 2021).

## A potential energy harvesting device made from recycled PET

The typical conversion processes from plastic waste to energy could be derived from three different approaches, namely chemical, biochemical and thermochemical. Lately, researchers have concentrated on increasing efficacy in power generation units for minimizing global warming. Integration of clean and renewable energy frameworks can concurrently reduce environmental pollution, enhance energy efficiency and reduce the total cost (Sharma et al. 2021).

**Fig. 10:**
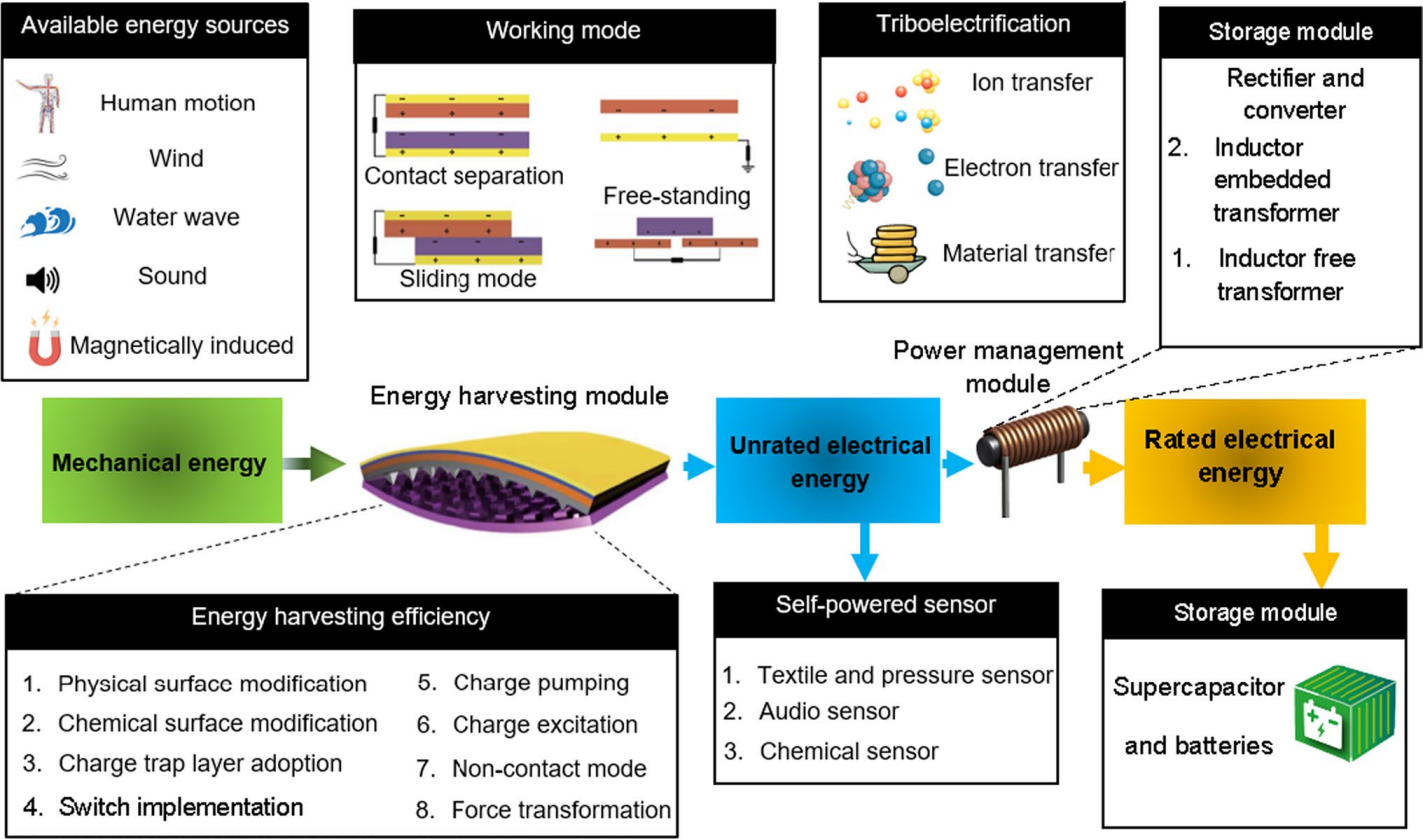
Block diagram of a TENG-based energy harvesting device

This section focus on a novel PET aerogel fabric that could be manufactured from recycled PET materials (Duong et al. 2021; Roy et al. 2021). A PET aerogel fabric made from the electrospun PET nanofibers could confer lightweight property, high porosity (large surface-to-volume ratio), high flexibility, excellent absorption capacity and low thermal conductivity on the aerogel (Thai et al. 2019). The excellent properties of PET aerogels have led to numerous potential applications, ranging from heat and sound insulations to wastewater treatment (Koh et al. 2018; Le et al. 2019, 2020; Thai et al. 2019). This focused discussion is on the use of PET aerogels for triboelectric nanogenerator (TENG), a device which is intended for sustainable energy production (see Fig. 10) (Roy et al. 2021). The use of recycled PET for making the aerogel has been proposed recently (Roy et al. 2021). The fabrication of recycled PET aerogels has been reported in several published articles (Le et al. 2019; Duong et al. 2021; Roy et al. 2021). The mesoporous sol–gel materials (70–90%) with large specific surface area (500–1000 m^2^/g), exceptional acoustic properties and low density (0.0001–0.200 g/cm^3^) are categorized as super insulators attributable to their ultralow thermal conductivity (12 mW/mK).

### Working principle of TENG

The working principle of TENG is based on triboelectrification, a type of contact electrification (see Fig. 10) by which the surface contacted between two surfaces causes the charges to travel from one side to another, generating significantly high voltages. The generation of electrical energy from mechanical energy relies on electrostatic induction and triboelectrification (Kim et al. 2021). Initially, the physical contact by one pair of triboelectric layers with distinct electron affinities results in the fabrication of charges. Second, external mechanical forces (derived from the range of natural sources, for instance, wind, rain or ocean waves to bodily motions including walking, running or finger movement) trigger the relative motion between triboelectric layers, breaking the balanced electrostatic charge distribution on the electrodes. Consequently, the electric potential difference built up between the electrodes is sufficient to trigger the free electrons to flow through the external circuits to establish an equilibrium. As the triboelectric layers return to their original position, the free electrons flow back to establish equilibrium (Zi et al. 2016). The alternating current arising from the oscillatory mechanical motions can be stored as energy in the storage unit or can be utilized for powering electric devices (Kim et al. 2020a).

### Performance of TENG

**Fig. 11:**
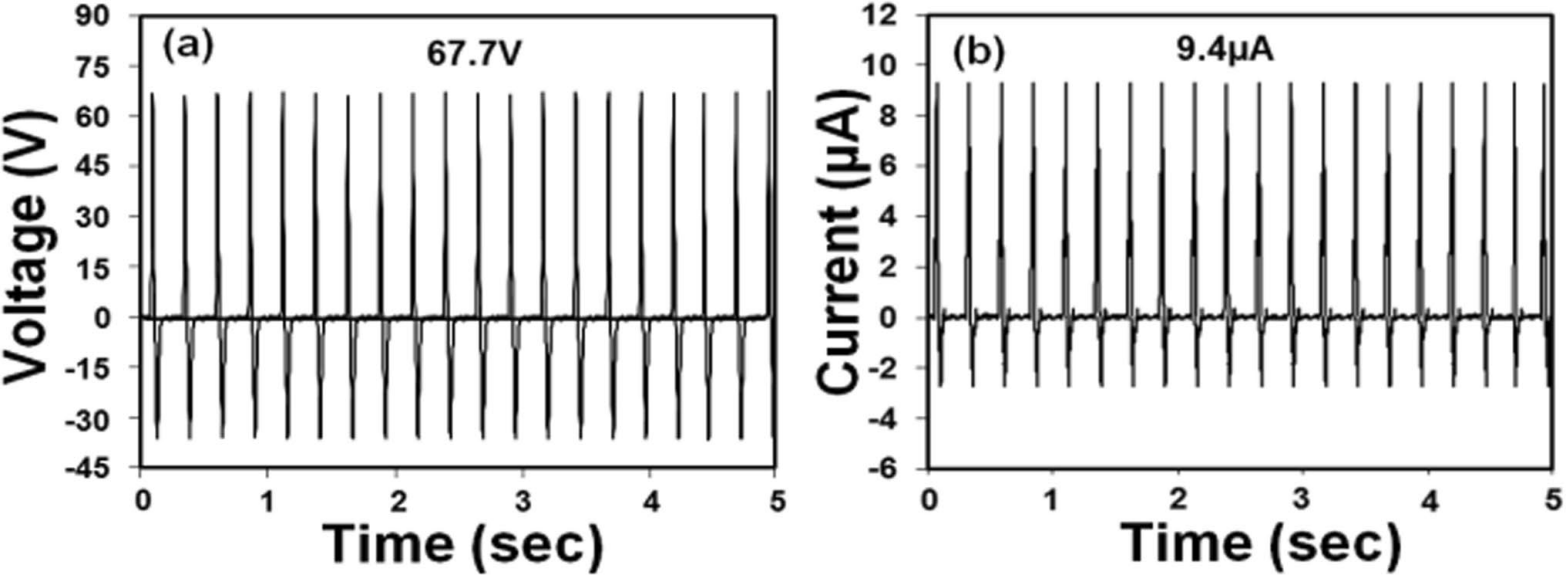
Triboelectric output **a** voltage and **b** current of TENG (Roy et al. 2021)

Figure 11 presents the triboelectric output (a) voltage and (b) current of the TENG in a recent study (Roy et al. 2021). The TENG (tribolayer size approx. 2 cm by 1 cm) could generate an output power of 636.4 μW (voltage ≈ 67.7 V; current ≈ 9.4 μA), enabling it to lit up 36 pieces of LEDs instantly. The TENG shows highly stable output performance over 10,000 cycles of continuous loading/unloading in room temperature over a period of 65 days with high-performance accuracy of about 99% (Roy et al. 2021).

The choice of material is important for improving the triboelectric effect and for procuring TENG with high output. There is a limited range of materials that have the desired electrical and mechanical properties such as breathability, washability, durability, lightness, extensibility and flexibility for use in TENG. The triboelectric series is a list of materials that rank according to their tendency to positive or negative charge used to select suitable pair of materials with opposite tribopolarity (Dzhardimalieva et al. 2021). The power generation in TENG is the result of the coupling effect of electrostatic induction and electrification. Therefore, ameliorating the contact charge generation on two dissimilar materials (compliant polymer ranked lower and the conducting polymer ranked higher in the triboelectric series) should be the most effective and inherent strategy to enhance the energy output (Feng et al. 2021). Furthermore, optimization of structural design of the device, such as increasing the contact area and functionalization of surface, could enhance the performance of the TENG device as reported in other published work (Mallineni et al. 2017; Zou et al. 2021).

**Fig. 12:**
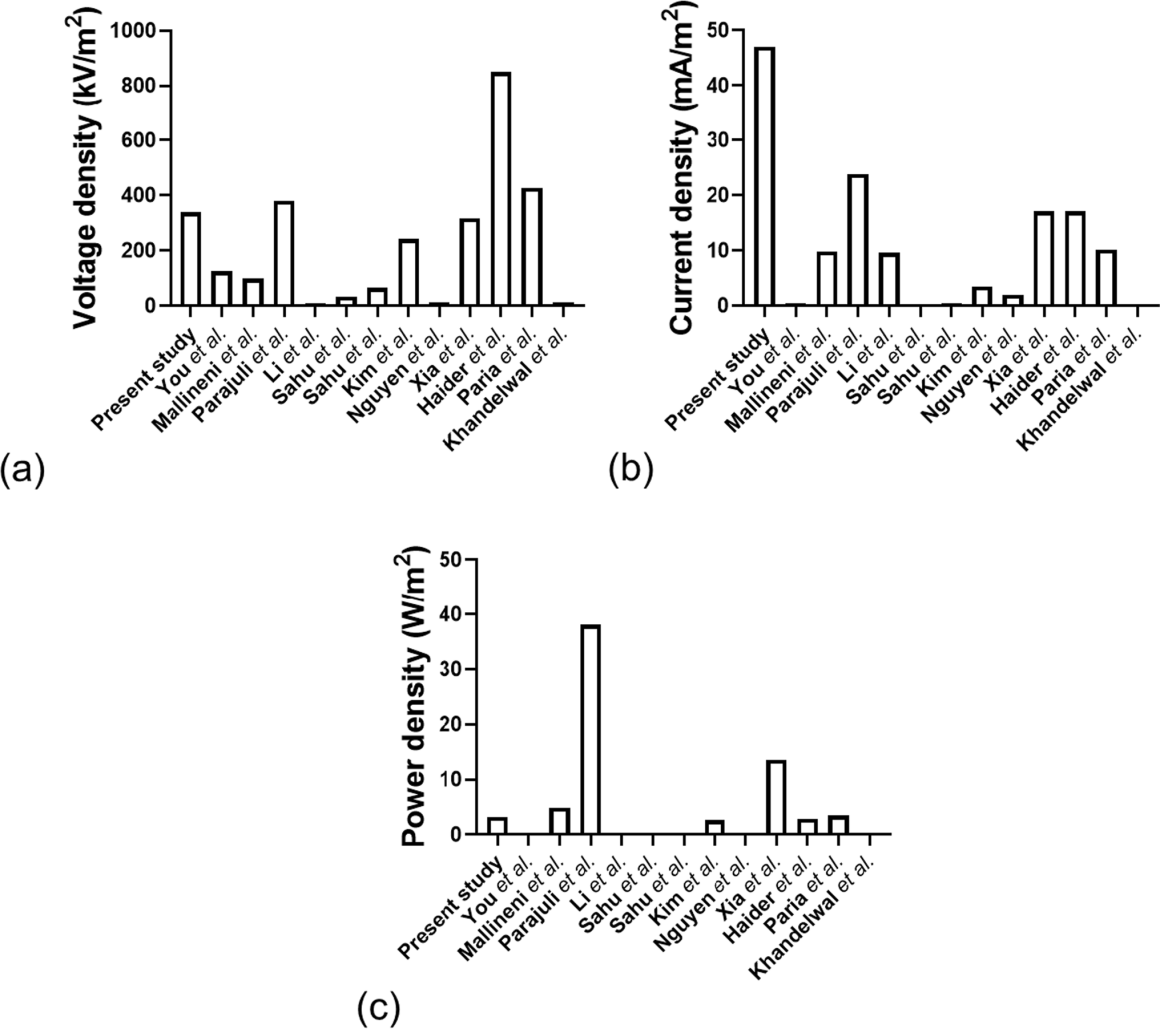
Comparison of the electrical output generated from TENG device in different studies. **a** Voltage density. **b** Current density. **c** Power density

Figure 12 shows the comparison of electrical output (namely voltage, current and power) of the TENG derived from various studies. In general, the findings from the studies showed that TENG could emit up to 200 colourful LEDs and able to power up a digital watch. Furthermore, cyclic mechanical loading was conducted to evaluate the durability and the reliability of the TENG. The findings showed that the TENG could be mechanically loaded for more than 10,000 cycles with a high performance accuracy of 99% (in the output voltage and current). In comparison among the different studies, the findings revealed that the performance of the TENG depends on a number of factors, namely (1) types of tribolayer materials, (2) types of surface modification on the tribolayers, (3) the size and density of the tribolayers and (4) the mode of mechanical motions and loading parameters (load and speed) to harvest energy (Bukhari et al. 2022). Therefore, interpreting and comparing the performance of the TENG from the different studies must be conservative. For more details on the comparison study on the performance of the TENG, refer to Table 7.

**Table 7:** Comparison of proposed TENG with other waste-derived TENG devices previously reported

Reference	Tribolayer 1	Tribolayer 2	Modification	Voltage (V)	Current (μA)	Power density (mW/m^2^)	Features
Present study (Roy et al. 2021)	Recycled PET	PVDF	Coated with polydopamine	67.7	9.4	3181.9	• Illuminate 36 colourful LEDs • Frequency: 4 Hz • 10,000 working cycles • Demonstrated remarkably high capability in removing various heavy metals such as Zn(II), Pb(II) and Hg(II) from the contaminated water with adsorption capacity between 94.5 and 98.3% • Size: 1 cm by 2 cm
You et al. (2021)	PET nanofibres within polydimethylsiloxane (PDMS) elastomer	Poly(methacrylate)	Coated with Ag	50	200,000	25	• Area: 4 cm^2^ • Ultrathin (0.30 mm) • Size and shape change according to application • Excellent flexibility • Tensile strength: 5.15 MPa • Elongation at a break of 270% • Frequency: 0.5 Hz
Mallineni et al. (2017)	PET	Indium tin oxide (ITO)	Nil	500	50	4902	• Illuminated 200 commercial LEDs • Powered a 8-digit handheld calculator in real time • 20,000 + working cycles • Stability at wide range of temperatures up to 60 °C • Frequency: 2 Hz
Parajuli et al. (2020)	PET	ITO	Fullerene modified	1600	100	38,095.2	• Powered a digital watch in real time Frequency: 2 Hz
Li et al. (2021)	Milk carton waste (Polyethylene and aluminium)	Copper electrode	Nil	9	8.64	86.4	• Efficient energy harvesting (charge density shift from 0.035 mC m − 2 to 1.00 mC m − 2 by integration of a charge excitation circuit) • 100,000 + working cycles • Frequency: 2.5 Hz
Sahu et al. (2021)	Laboratory waste (cotton, aluminium, glass, and nitrile gloves)	Waste PET	Nil	115	0.74	24.7	• Thickness (Al: 0.01 mm, nitrile glove: 0.22 mm, tissue paper: 0.19 mm, cotton: 0.65 mm, glass: 1.20 mm, PET: 0.26 mm) • Frequency: 2 Hz
Sahu et al. (2021)	Laboratory waste (cotton, aluminium, tissue paper, glass and nitrile gloves)	Waste plastic (mixed)	Nil	185	1.25	81.1	• Thickness (Al: 0.01 mm, nitrile glove: 0.22 mm, tissue paper: 0.19 mm, cotton: 0.65 mm, glass: 1.20 mm, plastic: 0.24 mm) • Frequency: 2 Hz
Kim et al. (2020b)	Polyimide on ITO-PET	Aluminium	Nil	753	10.79	261.2	• 10,000 working cycles • Illuminated 55 + LEDs • Frequency: 2 Hz
Nguyen et al. (2014)	Waste plastic bag (nylon, polyvinyl(chloride) (PVC), polyethylene (PE))	Gold	Nil	35.7	5.85	72	• Frequency: 5 Hz
Xia et al. (2019)	Tea leaves and Polytetrafluoroethylene (PTFE) film or aluminium plastic bags (PE/PET layer and PVC layer)	Aluminium	Nil	792	42.8	13,559	• Illuminating 179 green high-power LED • Shape: honeycomb lantern • 10,000 working cycles • Size: 5 cm by 5 cm • Frequency: 5 Hz
Lopez et al. (2021)	Polyethylene	Polycarbonate	Surface charge engineering	215.3	80	1,722,400	• 460% increase in output power after surface charge modification • Human walking test resulted in output of 16 V • Skateboard test (real time) resulted in output of 30 V • Truck test (real time) resulted in highest output of 60 V
Haider et al. (2020)	Cryogel (lauryl acrylate)	PDMS	Cross-linkers	170	3.42	2907	• Illuminate 180 white LEDs • Porosity: 73% • Average pore size: 2.6276 μm • Frequency: 7 Hz • Size: 1 cm by 2 cm
Paria et al. (2018)	Cigarette wrapper	Poly(vinylidene fluoride) (PVDF)	Nil	342	8.1	3462.8	• Illuminate 136 commercial LEDs • Was able to power up a digital hydrometer, wristwatch, and mobile LCD screen • 193,200 working cycles • Volume: 6.6 cm^3^ • Mass:1.259 g • Size: 4 cm by 2 cm • Frequency: 3.4 Hz
Khandelwal et al. (2018)	Mixed household plastic (PET bottles, polyethylene, polypropylene)	Polyurethane foam, polyethylene and polystyrene	Nil	44	289,000	3.2	• Illuminated 5 LEDs • Was used for intrusion monitoring for real-time testing • Detected small dynamic forces (2-12 kPa) with excellent sensitivity (10.92 nA kPa^–1^) • Fabrication time: 5 min • 3000 working cycles

### Benefits and limitations of TENG: production and deployment

Table 8 presents the benefits and limitations of TENG. There are many technology-related benefits of TENG (Liu et al. 2019; Yu et al. 2019; Wang et al. 2020). The TENG can be operated by many modes of mechanical motions (namely contact-separation mode, sliding mode, single-electrode mode and free-standing mode) which provides great accessibility to integrate TENG in the design for many applications (Godwinraj and George 2021). The TENG is a self-sustainable energy system that generates its own power source without requiring external resources; in other words, the TENG can be used to replace the battery in electronic devices (Godwinraj and George 2021).

**Table 8:** Benefits and limitations of TENG

Benefits	Limitations
Technology	
• Accessible design for different applications • Sustainable energy source • Able to generate electrical energy by harvesting using mechanical motion • High-energy efficiency to replace the battery for a low-powered electronic device (i.e. wearable device like watch or health tracker)	• Short product life due to decay of materials (wear and tear) caused by mechanical motions • Small amount of electrical energy could be harvested, depends on the size of the TENG
Environment	
• Alleviates the amount of PET waste by producing the aerogel using recycled PET materials • Innovation and invention of technology by using PET waste • Generating clean energy using recycled plastic resources • Reduction of plastic waste dumping into the landfill which impacted the ecology due to greenhouse gas emission and production of toxic substances	• Increase in the amount of wastewater/solvent generated to treat the recycled PET flakes to produce PET nanofibers during aerogel manufacturing • Increase in electrical energy consumption by the machineries to manufacture TENG • Increase disposal of TENG device at the end-of-life

The benefits and limitations of TENG can be categorized into two aspects, namely technology and environment. From technological perspectives, the benefits of TENG includes high voltage characteristics which makes TENG suitable for applications requiring high voltage requirements such as plasma generators and electric guns (Li et al. 2017; Cheng et al. 2018; Wang et al. 2020), while the low current characteristics of TENG limits their application for transferring power to storage devices. Furthermore, TENG has also found applications in self-powered sensor units in chemical and biological fields due to its ability to furnish power to devices (Kim et al. 2021). The limitation of the TENG includes short product life due to the decay of materials which mainly arises from wear and tear caused by the mechanical motions (Godwinraj and George 2021). As observed in several different studies (see Table 7), mechanical testing has been conducted to evaluate the performance and reliability of the TENG by consecutively loading the device. From a recently published study, the TENG was mechanically loaded for 65 days (about 10,000 cycles), the findings showed that the TENG did not failed and could produce a stable electrical output with accuracy up to 99% (Roy et al. 2021). Presently, the working/operational life of the TENG is uncertain, further mechanical testing on the materials used to produce the TENG will be required; however, this is out of the scope of this study. Furthermore, the TENG size may possibly has an influence on the amount of electrical energy that the device can harvest as increasing contact surface between two materials may possibly increase the amount of charges travelling from one side to another, generating significantly high voltages. It is difficult to compare the performance of the TENG from different studies (see Table 7), as the TENG varies in material types, material density and porosity, and sizes of devices.

In the aspect of the environment, the benefits of producing and deploying TENG may alleviate the amount of PET waste and energy problem in rising economic growth. The implementation of recycled PET materials as feedstock for manufacturing aerogels could reduce the volume of PET plastic waste going into the incinerator and dumping into the landfill. The usage of PET waste could boost the recycling rate of both Singapore and India by keeping the plastic resources in the closed-circular economy. The environment-related limitations for manufacturing TENG include increasing of wastewater or solvent used to treat the recycled PET flakes for PET aerogel manufacturing and an increase in electrical consumption by machineries during TENG manufacturing. Improper treatment and disposal of wastewater/solvent lead to environmental pollution resulting in toxic to human, terrestrial acidification and marine/freshwater eutrophication (Vollmer et al. 2020). Another limitation to consider is the end-of-life of TENG, and how the TENG could be recycled. Disposal of TENG leads to increasing of waste which may possibly strain the waste treatment processes. No further analysis on the environmental impact assessment and the end-of-life of the TENG product are required for our present purpose; however, it is an important area to study when performing the life-cycle analysis for manufacturing of TENG product.

### Challenges of using plastic scraps for manufacturing

**Fig. 13:**
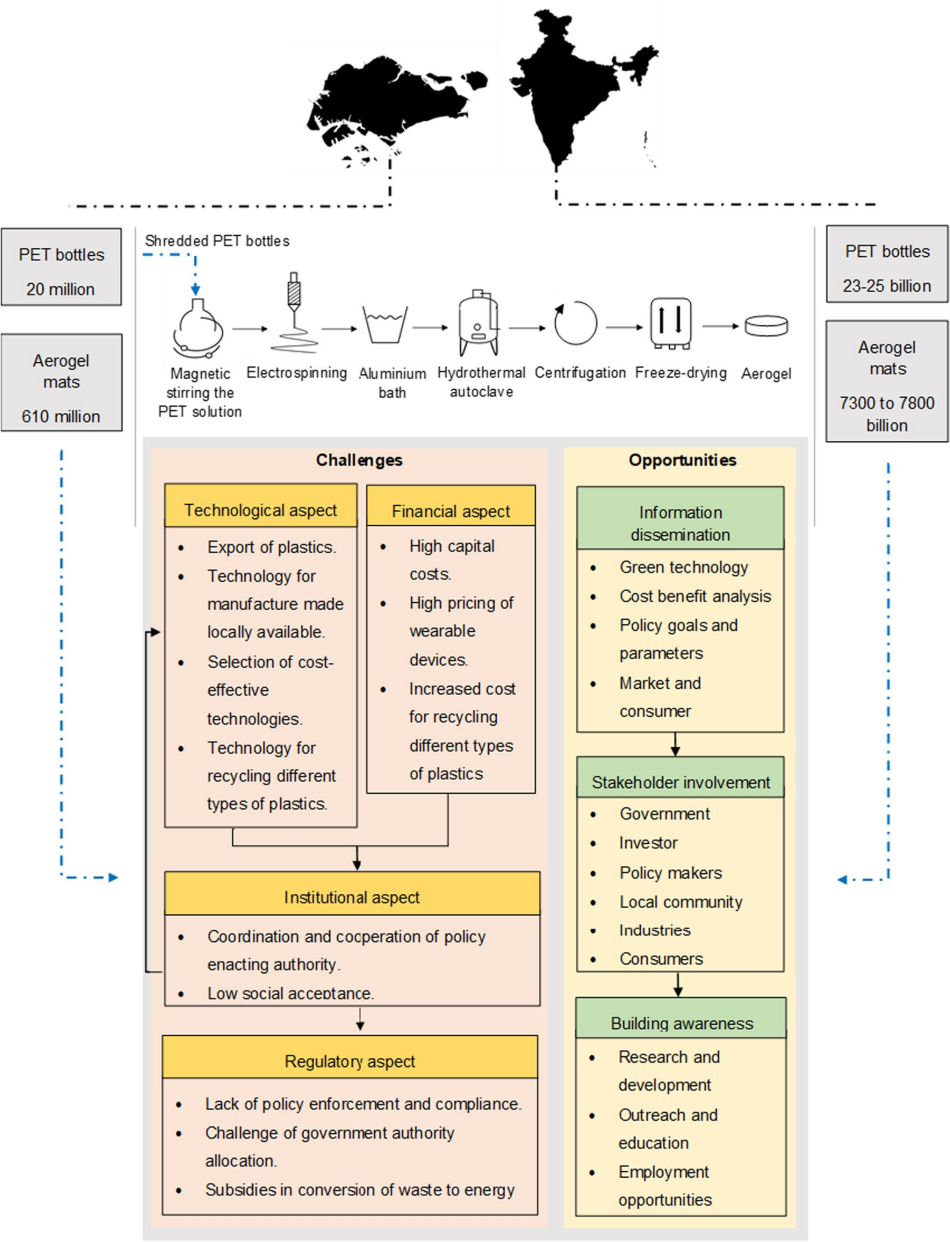
In-country supply chain challenges and opportunities for plastic waste to TENG

TENG has many remarkable merits (as shown in Table 8); however, in-country supply chains must be established in order to commercialize and market the TENG devices. Infrastructures for a sustainable supply chain need to be put in place and their activities include recycling of plastic scraps, manufacturing, storage, packaging, transportation and to end-users/consumers (Mc Loughlin et al. 2021). Evidently, the current price of producing recycled plastics was high (due to the effort required for managing and costs of recycling processes) which makes wider adoption as feedstock for manufacturing to be difficult (Arwa Mahdawi 2020). In-country supply challenges and integrated opportunities for futuristic sustainable development in converting plastic waste to TENG are embellished through Fig. 13.

In Singapore, the supply of recycled plastic scraps for manufacturing of TENG devices poses a challenge as most of the plastic scraps were exported to neighbouring countries for recycling (Kerdlap et al. 2020, 2021). In a recent report, the plastic recycling association of Singapore envisioned to build its first operational plastic recycling plant in the country by the year 2023 and aims to increase the plastic recycling rate from 4 (in 2020) to 30% in the next 5 years to support the Singapore Green Plan 2030, a national movement to advance Singapore’s national agenda on sustainable development (Justin Ong 2021). The report showed that an estimate of 500 million PET bottles was disposed yearly and only 4% were recycled (about only 20 million PET bottles) to check if the recycled materials would be sufficient to achieve a sustainable manufacturing of TENG devices in Singapore. For the purpose of illustration, an order of magnitude estimates are considered for an everyday wearable application, namely the digital wristwatch (as an alternative to existing solar-powered ones) as highlighted in a previously published report by Wang et al. (2017a). One 500-ml PET bottle results in aerogel of size 210 mm in length by 297 mm in width (Crystal Ho 2018). Considering 20 million PET bottles (of 500-ml PET bottles) were recycled in 2020, an estimation of about 610 million pieces of aerogel mats (size: 20 mm in length by 10 mm in width) could be produced. When the recycling rate increased to 30% (about 150 million PET bottles) in the next 5 years, an estimated of about 46.5 billion pieces of aerogel mats could be produced. However, India is able to house all the recycling processes and facilities in the country (Ministry of Housing and Urban Affair 2019). An approximately 900,000 tonnes of PET bottles were disposed, and 23 to 25 billion pieces of PET bottles (~ 800,000 tonnes) were recycled (recycling rate = 90% for PET waste) yearly since 2017. Here, an estimation of about 7300 to 7800 billion pieces of aerogel mats (size: 20 mm in length by 10 mm in width) could be produced. Based on the findings, i.e. the amount of recycled PET plastics that were disposed yearly, if a proportion of this were to be used to make PET aerogels for the TENG devices, it would be sufficient to produce small-scale TENG wearable devices to cater for all the residents/peoples in Singapore (population ≈ 5.68 million in 2020) and India (population ≈ 1.38 billion in 2020).

Herein, the estimates of the recycled PET plastics used for manufacturing one tribolayer of aerogel of the TENG device were evaluated. The usage of PET plastic scraps could only recover a small percentage (about 4% for Singapore and 1.5% for India) of the PET scraps from the total amount of plastic waste generated in the respective countries. However, recovery of different plastic types from the generated waste is required in order to have a significant change in the plastic waste recovery system. Likewise, apart from PET, other types of plastic scraps (i.e. polyethylene, polycarbonate, polystyrene, polyvinyl chloride) could also be used to make different components of the TENG device such as an alternative tribolayers, device casing and packaging. However, employing different recycled plastic materials to produce TENG device is a challenge as the different material characteristics may affect the performance of the TENG (see Table 7). Furthermore, the recycling processes for different plastic scraps may differ due to the different in the resin characteristics (Rick Leblanc 2020). Additional recycling processes for different types of plastic scraps may increase the costs of the recycled plastics which increases the difficulty for the manufacturer to accept recycled plastics materials in production (Rick Leblanc 2020). Further studies are important to study the recycling processes for different plastic scraps and to evaluate the performance of the TENG using different recycled plastic materials to explore their benefits for different applications. However, the studies were beyond the scope of this paper.

Moreover, another big challenge is the low recycling rates due to the lack of improved technologies in plastic waste collection, sorting and segregation and the lack of authentic data about recyclable and recycled plastics (Woidasky et al. 2020). Large-scale production and commercialization of TENG would demand a secured platform for tagging, testing and tracking the plastic products to measure the real value of plastic products in terms of its recyclability. Advanced technology with unique molecular tags (molecular barcodes) applied in the manufacturing process may help in solving the issue of ambiguous plastic labelling (Sandhiya and Ramakrishna 2021). Blockchain technology, a digital innovation, offers the potential to trace the journey of plastic products across their life cycle from production, manufacturing, usage and disposal. These records can be further utilized by different economic agents, serving as a trust-based platform between plastic waste segregators, recyclers and recycled feedstock buyers (Chidepatil et al. 2020; Liu et al. 2021). As per the strategy of blockchain technology, stakeholders are benefitted for their involvement in the validation process using digital cash (Sandhiya and Ramakrishna 2021). The shortcomings of the present sorting and segregation technologies may be overcome with the application of a multi-sensor driven artificial intelligence approach, i.e. using high-definition optical sensors to identify the shape, colour and texture of plastics to separate selected recyclables or incompatible materials in the recycling process (Chidepatil et al. 2020; Gussen et al. 2020).

## Conclusions

Efficacious plastic waste management demands the highest priority from the environmental and economic perspectives. Comparing the two large economies (Singapore and India) based on governance, social, market regulatory and financial data, the study evaluated how both countries managed their waste system. The findings were summarized as follows,

1. The legislation and policy measures have been implemented in both Singapore and India to administer regulatory functions of waste collection, treatment and disposal for identifying the key waste stream and strengthen the resource resilience.
2. Recycling infrastructure (i.e. MRF, WTE plant and landfill) and technologies (i.e. mechanical recycling, pyrolysis and incineration) have been developed in both countries to convert the waste to useful resources. The recycling statistics showed that the plastic recycling rate in Singapore were much lower (most were being incinerated) as compared to India.
3. The economic value of recycled PET materials was estimated for considering as resources for remanufacturing. It was predicted that the economic value of PET depreciates after each consecutive recycling cycle as compared to virgin PET materials. Also, the economic value of the PET depreciates when the mechanical performance of the PET materials reduces after each recycling cycle.
4. An emerging technology known as TENG, which is an energy harvesting device, was proposed. The production of TENG using recycled plastics could appreciably aid in reducing plastic waste and keeping the plastic resources in the closed-loop economy, thus promoting sustainable cities and communities. Rerouting waste streams and utilizing them as chemical feedstocks should become pervasive for the manufacturing of merchantable products to accomplish the complete and continuous circulation of resources within the circular economy.

In addition, this work had benefitted from productive collaboration at the academic level and cooperation between industry and academic researchers, non-government entities and legislative bodies.

## Supplementary Information

Below is the link to the electronic supplementary material.

Supplementary file1 (DOCX 35 KB)

## Data Availability

The data are available in the Supplementary Information document.

## Abbreviations

3Rs: Reduce, reuse, recycle
CPCB: Central pollution control board
EPHA: Environment public health act
EPR: Extended producer responsibility
GDP: Gross domestic product
GWC: General waste collector
HDB: Housing development board
HDPE: High-density polyethylene
LCA: Life cycle analysis
LDPE: Low-density polyethylene
MoEF: Ministry of environment, forest, and climate change
MPR: Mandatory packaging reporting
MRF: Materials recovery facilities
NEA: National Environment Agency
PA: Polyamide
PBAT: Polybutylene adipate terephthalate
PBS: Polybutylene succinate
PE: Polyethylene
PET: Polyethylene terephthalate
PHA: Polyhydroxyalkanoates
PLA: Polylactic acid
PP: Polypropylene
PPP: Surface-modified PET aerogel
PRO: Producer responsibility organization
PS: Polystyrene
PTT: Polytrimethylene terephthalate
PVA: Polyvinyl alcohol
PVC: Polyvinyl chloride
PVDF: Polyvinylidene fluoride
PWC: Public waste collector
PWM: Plastic waste management rules
RVM: Recyclate value model
SBM: Swachh Bharat mission
SEC: Singapore Environment Council
SPCB: State Pollution Control Board
TENG: Triboelectric nanogenerator
WRMS: Waste and Resource Management System
WTE: Waste to energy
