# Supplementary material for: Roadmap to sustainable plastic waste management: a focused study on recycling PET for triboelectric nanogenerator production in Singapore and India
Source: Environ Sci Pollut Res Int. 2022 May 23;29(34):51234–68. doi: 10.1007/s11356-022-20854-2 (PMC9125019; doi:10.1007/s11356-022-20854-2)
Supplement: Supplementary file 1 — Supplementary file1 (DOCX 35 KB) [file 11356_2022_20854_MOESM1_ESM.docx]

**Roadmap to sustainable plastic waste management: a focused study on recycling PET for triboelectric nanogenerator production in Singapore and India**

Wei Liang Lai, Shreya Sharma, Sunanda Roy, Pradip Kumar Maji, Bhasha Sharma, Seeram Ramakrishna, Kheng Lim Goh

Supplementary Section 1: Economic values of PET plastic.

Table S-1. Details used to derive and calculate for the recyclate value of PET plastic.

| **Region** | **No. of times recycled?** | **Currency** | **Estimated economic value, RV_i-1_** | **Retained mechanical performance factor, m** | | **Reclaimed recyclate, r_p_** | **Recycling cost, P (USD)** | **Recyclate value, RV_i_ (USD)** | **Reference of mechanical performance** |
| --- | --- | --- | --- | --- | --- | --- | --- | --- | --- |
|  |  |  | **(USD/mt)** | **(0 ≤ m < 1)** | |  |  |  |  |
|  |  |  |  | **Rupture strain (%)** | **Factor** | **(0 ≤ r_p_ < 1)** |  |  |  |
| **Enlongation at break (%), ε_B_** | | | | | | | | | |
| South-East Asia (Singapore) | 0 | USD | 1341 | 42 | 1.000 | 1 | 50 | 1291.00 | Del Mar Castro López M, Ares Pernas AI, Abad López MJ, Latorre AL, López Vilariño JM, González Rodríguez MV. Assessing changes on poly(ethylene terephthalate) properties after recycling: Mechanical recycling in laboratory versus postconsumer recycled material. Mater Chem Phys 2014;147:884–94. https://doi.org/10.1016/j.matchemphys.2014.06.034. |
|  | 1 |  | 610 | 35 | 0.833 | 0.97 |  | 443.08 |  |
|  | 2 |  | 427 | 5.7 | 0.136 | 0.94 |  | 4.47 |  |
|  | 3 |  | 299 | 2.6 | 0.062 | 0.91 |  | -33.16 |  |
|  | 4 |  | 209 | 1.6 | 0.038 | 0.88 |  | -42.99 |  |
|  | 5 |  | 146 | 0.7 | 0.017 | 0.85 |  | -47.93 |  |
| South Asia (India) | 0 | USD | 1090 | 42 | 1.000 | 1 | 50 | 1040.00 | Del Mar Castro López M, Ares Pernas AI, Abad López MJ, Latorre AL, López Vilariño JM, González Rodríguez MV. Assessing changes on poly(ethylene terephthalate) properties after recycling: Mechanical recycling in laboratory versus postconsumer recycled material. Mater Chem Phys 2014;147:884–94. https://doi.org/10.1016/j.matchemphys.2014.06.034. |
|  | 1 |  | 680 | 35 | 0.833 | 0.97 |  | 499.67 |  |
|  | 2 |  | 476 | 5.7 | 0.136 | 0.94 |  | 10.72 |  |
|  | 3 |  | 333 | 2.6 | 0.062 | 0.91 |  | -31.24 |  |
|  | 4 |  | 233 | 1.6 | 0.038 | 0.88 |  | -42.19 |  |
|  | 5 |  | 163 | 0.7 | 0.017 | 0.85 |  | -47.69 |  |
| **Impact strength (kJ/m2) , α_cU_** | | | | | | | | | |
| South-East Asia (Singapore) | 0 | USD | 1341 | 135 | 1.000 | 1 | 50 | 1291.00 | Del Mar Castro López M, Ares Pernas AI, Abad López MJ, Latorre AL, López Vilariño JM, González Rodríguez MV. Assessing changes on poly(ethylene terephthalate) properties after recycling: Mechanical recycling in laboratory versus postconsumer recycled material. Mater Chem Phys 2014;147:884–94. https://doi.org/10.1016/j.matchemphys.2014.06.034. |
|  | 1 |  | 610 | 71 | 0.526 | 0.97 |  | 261.19 |  |
|  | 2 |  | 427 | 25 | 0.185 | 0.94 |  | 24.33 |  |
|  | 3 |  | 299 | 16 | 0.119 | 0.91 |  | -17.75 |  |
|  | 4 |  | 209 | 6 | 0.044 | 0.88 |  | -41.83 |  |
|  | 5 |  | 146 | 6 | 0.044 | 0.85 |  | -44.48 |  |
| South Asia (India) | 0 | USD | 1090 | 135 | 1.000 | 1 | 50 | 1040.00 | Del Mar Castro López M, Ares Pernas AI, Abad López MJ, Latorre AL, López Vilariño JM, González Rodríguez MV. Assessing changes on poly(ethylene terephthalate) properties after recycling: Mechanical recycling in laboratory versus postconsumer recycled material. Mater Chem Phys 2014;147:884–94. https://doi.org/10.1016/j.matchemphys.2014.06.034. |
|  | 1 |  | 680 | 71 | 0.526 | 0.97 |  | 296.90 |  |
|  | 2 |  | 476 | 25 | 0.185 | 0.94 |  | 32.86 |  |
|  | 3 |  | 333 | 16 | 0.119 | 0.91 |  | -14.09 |  |
|  | 4 |  | 233 | 6 | 0.044 | 0.88 |  | -40.89 |  |
|  | 5 |  | 163 | 6 | 0.044 | 0.85 |  | -43.84 |  |
| **Viscosity (Pa.s), η** | | | | | | | | | |
| South-East Asia (Singapore) | 0 | USD | 1341 | 350 | 1.000 | 1 | 50 | 1291.00 | Schyns ZOG, Shaver MP. Mechanical Recycling of Packaging Plastics: A Review. Macromol Rapid Commun 2021;42:1–27. https://doi.org/10.1002/marc.202000415.  La Mantia FP, Vinci M. Recycling poly(ethyleneterephthalate). Polym Degrad Stab 1994;45:121–5. https://doi.org/10.1016/0141-3910(94)90187-2. |
|  | 1 |  | 610 | 300 | 0.857 | 0.97 |  | 457.17 |  |
|  | 2 |  | 427 | 180 | 0.514 | 0.94 |  | 156.42 |  |
|  | 3 |  | 299 | 150 | 0.429 | 0.91 |  | 66.61 |  |
|  | 4 |  | 209 | 130 | 0.371 | 0.88 |  | 18.31 |  |
|  | 5 |  | 146 | 110 | 0.314 | 0.85 |  | -11.00 |  |
| South Asia (India) | 0 | USD | 1090 | 350 | 1.000 | 1 | 50 | 1040.00 |  |
|  | 1 |  | 680 | 300 | 0.857 | 0.97 |  | 515.37 |  |
|  | 2 |  | 476 | 180 | 0.514 | 0.94 |  | 180.11 |  |
|  | 3 |  | 333 | 150 | 0.429 | 0.91 |  | 79.87 |  |
|  | 4 |  | 233 | 130 | 0.371 | 0.88 |  | 26.16 |  |
|  | 5 |  | 163 | 110 | 0.314 | 0.85 |  | -6.46 |  |
| **Degree of crystallinity (%), D** | | | | | | | | | |
| South-East Asia (Singapore) | 0 | USD | 1341 | 21.5 | 1.000 | 1 | 50 | 1291.00 | Schyns ZOG, Shaver MP. Mechanical Recycling of Packaging Plastics: A Review. Macromol Rapid Commun 2021;42:1–27. https://doi.org/10.1002/marc.202000415.  La Mantia FP, Vinci M. Recycling poly(ethyleneterephthalate). Polym Degrad Stab 1994;45:121–5. https://doi.org/10.1016/0141-3910(94)90187-2. |
|  | 1 |  | 610 | 21.8 | 1.014 | 0.97 |  | 549.96 |  |
|  | 2 |  | 427 | 23 | 1.070 | 0.94 |  | 379.38 |  |
|  | 3 |  | 299 | 24 | 1.116 | 0.91 |  | 253.73 |  |
|  | 4 |  | 209 | 24.5 | 1.140 | 0.88 |  | 159.58 |  |
|  | 5 |  | 146 | 25.2 | 1.172 | 0.85 |  | 95.46 |  |
| South Asia (India) | 0 | USD | 1090 | 21.5 | 1.000 | 1 | 50 | 1040.00 |  |
|  | 1 |  | 680 | 21.8 | 1.014 | 0.97 |  | 618.80 |  |
|  | 2 |  | 476 | 23 | 1.070 | 0.94 |  | 428.66 |  |
|  | 3 |  | 333 | 24 | 1.116 | 0.91 |  | 288.27 |  |
|  | 4 |  | 233 | 24.5 | 1.140 | 0.88 |  | 183.65 |  |
|  | 5 |  | 163 | 25.2 | 1.172 | 0.85 |  | 112.39 |  |
